# Genomic Variations in the Tea Leafhopper Reveal the Basis of Its Adaptive Evolution

**DOI:** 10.1016/j.gpb.2022.05.011

**Published:** 2022-08-28

**Authors:** Qian Zhao, Longqing Shi, Weiyi He, Jinyu Li, Shijun You, Shuai Chen, Jing Lin, Yibin Wang, Liwen Zhang, Guang Yang, Liette Vasseur, Minsheng You

**Affiliations:** 1 State Key Laboratory of Ecological Pest Control for Fujian and Taiwan Crops, Institute of Applied Ecology, Fujian Agriculture and Forestry University, Fuzhou 350002, China; 2 Institute of Rice, Fujian Academy of Agricultural Sciences, Fuzhou 350018, China; 3 Tea Research Institute, Fujian Academy of Agricultural Sciences, Fuzhou 350001, China; 4 Center for Genomics and Biotechnology, Fujian Agriculture and Forestry University, Fuzhou 350002, China; 5 Department of Biological Sciences, Brock University, St. Catharines, ON L2S 3A1, Canada

**Keywords:** Tea green leafhopper, Genomic variation, Population genetics, Local adaptation, Evolutionary history

## Abstract

**Tea green leafhopper** (TGL), *Empoasca onukii*, is of biological and economic interest. Despite numerous studies, the mechanisms underlying its adaptation and evolution remain enigmatic. Here, we use previously untapped genome and **population genetics** approaches to examine how the pest adapted to different environmental variables and thus has expanded geographically. We complete a chromosome-level assembly and annotation of the *E*. *onukii* genome, showing notable expansions of gene families associated with adaptation to chemoreception and detoxification. Genomic signals indicating balancing selection highlight metabolic pathways involved in adaptation to a wide range of tea varieties grown across ecologically diverse regions. Patterns of genetic variations among 54 *E*. *onukii* samples unveil the population structure and **evolutionary history** across different tea-growing regions in China. Our results demonstrate that the genomic changes in key pathways, including those linked to metabolism, circadian rhythms, and immune system functions, may underlie the successful spread and adaptation of *E*. *onukii*. This work highlights the genetic and molecular basis underlying the evolutionary success of a species with broad economic impacts, and provides insights into insect adaptation to host plants, which will ultimately facilitate more sustainable pest management.

## Introduction

**Supplementary Figure S1:**
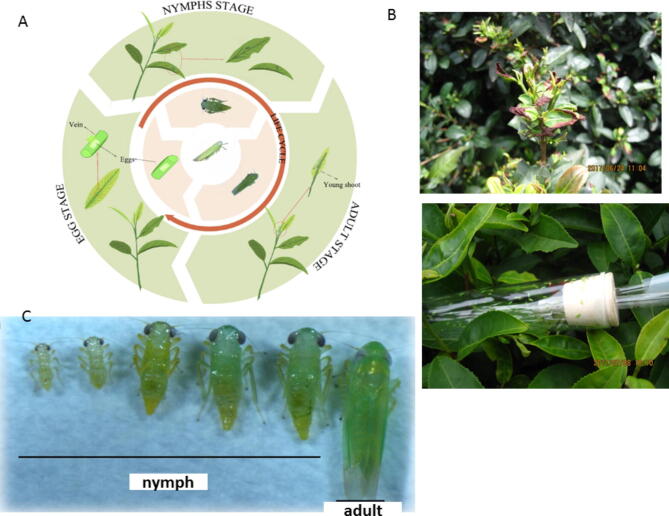
**Damages of *E. onukii* in modern tea plantation in China A.** Life cycle of *E. onukii*. **B.** Damages caused by *E. onukii*. **C.** Developmental stages of *E. onukii*.

Tea is the most popular beverage worldwide, surpassing coffee and cocoa, with a production of 6.1 million metric tons in 2019 (International Tea Committee; https://www.statista.com/statistics/264183/global-production-and-exports-of-tea-since-2004/). China represents the largest tea producer, consumer, and exporter in the world. In Asia, the tea green leafhopper (TGL), *Empoasca onukii* (Hemiptera: Cicadellidae), represents the most devastating pest across tea plantations, causing up to 50% economic loss of tea production annually [1], [2]. Both nymph and adult TGLs pierce and suck the sap of tender tea shoots, which are the most important part of the plant to produce high-quality tea. Adult females also lay their eggs in these shoots, leading to irreparable damage (Figure S1) [2], [3]. Presence of local TGL population has been recorded in China since the 1950s [4]. And the distribution has increased around tea-producing regions of China, Japan, and Vietnam [5]. *E*. *onukii* can cause yield loss of 15%–50%, up to 100% in severely damaged plantations [2], [6].

TGL belongs to the most species-rich hemimetabolous order, various species of which are agricultural pests or human disease vectors [7]. As a monophagous insect, TGL is well-adapted, both physiologically and biochemically, to different tea varieties [8]. Thus, the rapid expansion of *E*. *onukii* raises critical questions concerning which factors contribute to its successful dispersal and colonization, and how genomic architecture underlies its broad and rapid ability to adapt.

To address the aforementioned questions, we generated a chromosome-level genome assembly of *E*. *onukii* by integrating Illumina short reads, Oxford Nanopore Technologies (ONT) long reads, and high-throughput chromosome conformation capture (Hi-C) data. This high-quality genome resource enabled us to investigate the genetic basis of chemoreception and detoxification in this insect, key to adapting to new environments. Based on 54 resequenced genomes of the *E*. *onukii* samples collected from different locations across a diverse range of tea-growing regions in China, we analyzed the patterns of genomic variation and population structure in this species, allowing us to gain insights into its evolutionary history as well as successful, rapid spread and colonization.

## Results and discussion

### Chromosome-level assembly of the TGL genome

**Figure 1:**
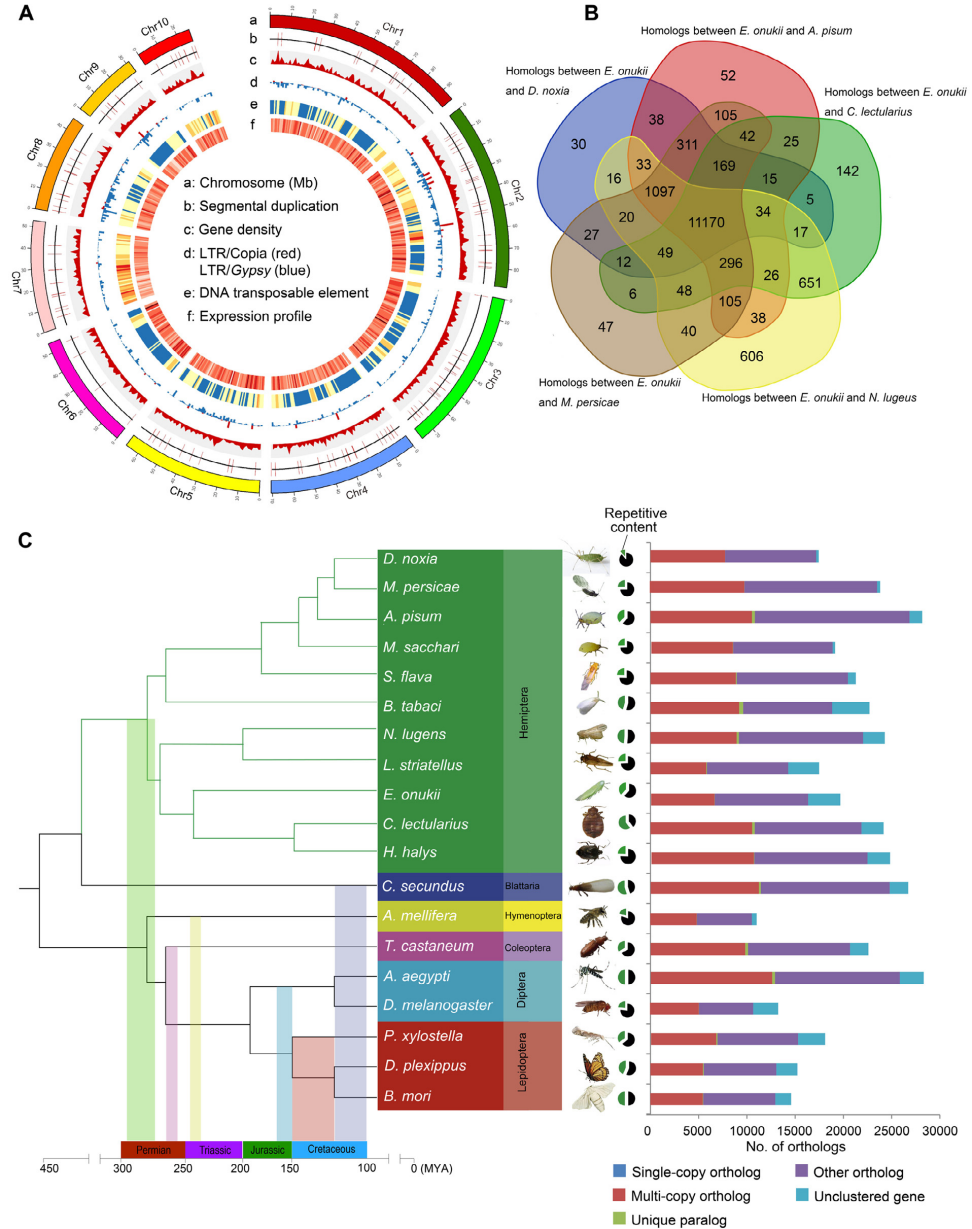
**Genomic characterization of *E***. ***onukii* and comparison with other insect genomes** **A.** Genomic characterization of the sequenced *E*. *onukii*. a, chromosome (Mb); b, segmental duplication; c, gene density; d, LTR/*Copia* (red) and LTR/*Gypsy* (blue); e, DNA transposable element; f, expression profile. **B.** Comparison of homologous genes between gene sets from *E*. *onukii* and each of the five Hemiptera species. Dataset overlaps were determined using a BLASTP search (E-value < 1 × 10^−^^5^). **C.** Phylogenetic relationships among 15 insect species based on genomic comparisons. Single-copy orthologs represent genes with only one copy in different genomes. Multi-copy orthologs represent genes with more than one copy in different genomes. Unique paralogs represent species-specific genes. Other orthologs represent unclassified orthologs. Unclustered genes represent genes that cannot be clustered into known gene families. Details about the identification are previously described [72]. Chr, chromosome; LTR, long terminal repeat; *D. noxia*, *Diuraphis noxia*; *M. persicae*, *Myzus persicae*; *A. pisum*, *Acyrthosiphon pisum*; *M. sacchari*, *Melanaphis sacchari*; *S. flava*, *Sipha flava*; *B. tabaci*, *Bemisia tabaci*; *N. lugens*, *Nilaparvata lugens*; *L. striatellus*, *Laodelphax striatellus*; *E. onukii*, *Empoasca onukii*; *C. lectularius*, *Cimex lectularius*; *H. halys*, *Halyomorpha halys*; *C. secundus*, *Cryptotermes secundus*; *A. mellifera*, *Apis mellifera*; *T. castaneum*, *Tribolium castaneum*; *A. aegypti*, *Aedes aegypti*; *D. melanogaster*, *Drosophila melanogaster*; *P. xylostella*, *Plutella xylostella*; *D. plexippus*, *Danaus plexippus*; *B. mori*, *Bombyx mori*.

**Supplementary Figure S2:**
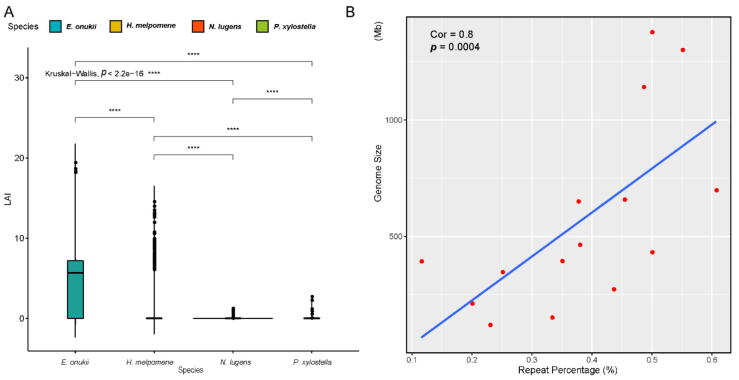
**Genome assembly of *E. onukii* A.** Chromatin interactions with 150-kb resolution in *E. onukii*. **B.** Mitochondrial genome of *E. onukii*. Inner circle represents the GC content whereas outer circle represents genes located on mitochondrion. ATP, adenosine triphosphate; NADH, nicotinamide adenine dinucleotide.

The genome of *E*. *onukii* was estimated to be ∼ 608 Mb based on *k*-mer analysis. We combined 61× Illumina short reads and 109× ONT sequences with chromosome-scale scaffolding. We informed our assembly using physical mapping of Hi-C (Tables S1 and S2), to generate an assembly based on 599 Mb of sequences, with the mitochondrial sequences excluded (Table 1, Tables S3 and S4). This assembly accounted for 98.5% of the estimated genome size. A total of 592-Mb sequences, covering 98.83% of the assembled genome, were then anchored onto 10 pseudo-chromosomes using the ALLHiC pipeline (see Materials and methods; Figure 1A, Figure S2A; Table 1, Table 2). An official gene set was generated based on alignment of insect gene homologs, *ab initio* predictions, and transcriptomic evidence. Genome annotation predicted 19,642 protein-coding genes with 92.5% Benchmarking Universal Single-Copy Orthologs (BUSCO) completeness in *E*. *onukii* (Table 1, Table S5). The sequenced *E*. *onukii* genome showed high heterozygosity (2.8%), with 13,122,207 heterozygous single nucleotide polymorphisms (SNPs), 3,796,369 heterozygous insertions and deletions (InDels), and complex segmental duplication patterns (Figure 1A). We also assembled the mitochondrial genome, which had a total length of 14.2 kb and 13 protein-coding genes annotated (Figure S2B).

**Table 1:** **Sequencing, chromosome-scale assembly, and annotation of the *E.onukii* genome**

**Sequencing**	***E. onukii***
Sequencing platform	ONT
Data size (Gb)	65
Genome sequencing depth (×)	109
Estimated genome size (Mb)	∼ 608
**Chromosome-scale assembly**	
Genome estimated (Mb)	608
Assembly size (Mb)	599
Percentage of estimated genome size (%)	98.5
No. of contigs	1800
Contig N50 (Mb)	2.2
Average length (bp)	332,835
Minimum contig length (bp)	2552
No. of chromosomes	10
Scaffold N50 (Mb)	67.98
No. of unanchored contigs	234
Length of unanchored contigs (Mb)	7
No. of anchored contigs	1566
Length of anchored contigs (Mb)	592
Anchor rate (%)	98.83
BUSCO completeness (%)	92.7
**Annotation**	
No. of protein-coding genes	19,642
Average gene length (bp)	7904
Average CDS length (bp)	201
Average exon number per gene	4.99
BUSCO completeness (%)	92.5

*Note*: ONT, Oxford Nanopore Technologies; BUSCO, Benchmarking Universal Single-Copy Orthologs; CDS, coding sequence.

**Table 2:** **Chromosome-based statistics of the *E*. *onukii* genome**

**Chromosome**	**No. of contigs**	**Length (bp)**
Chr1	263	94,216,414
Chr2	314	91,501,843
Chr3	121	74,646,503
Chr5	202	67,983,564
Chr4	155	65,611,511
Chr6	79	54,167,312
Chr8	177	48,146,360
Chr7	88	43,255,712
Chr9	110	28,073,217
Chr10	57	24,627,475

**Supplementary Figure S3:**
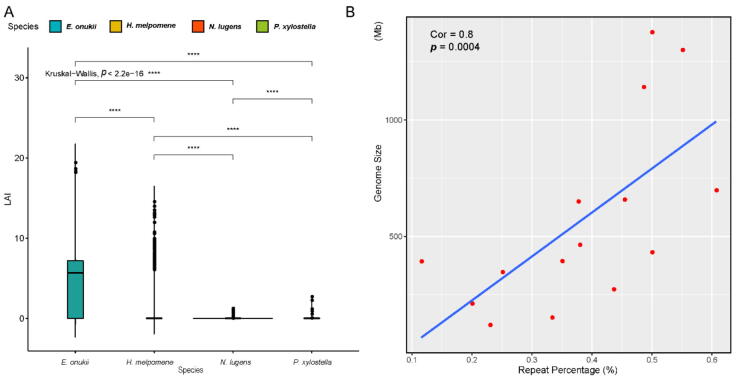
**Assessment of the assembly A.** Assessment of the assembly using LAI. **B.** Correlation analysis between repeat content and the genome size. LAI, LTR Assembly Index.

Compared with recently published Hemiptera genomes [9], [10], this assembly is a high-quality genome with 92.7% BUSCO completeness (Tables S4 and S6). Around 92.3% (337.5/365.8 million) of the Illumina short reads were mapped to the assembled reference, representing ∼ 94% of the genome (Table S7). Well-organized patterns of interacting contact along the diagonal for each pseudo-chromosome confirmed the high-quality chromosome-level assembly (Figure S2). In addition, assessment using the long terminal repeat (LTR) Assembly Index (LAI) [11] revealed that more intact LTRs were recalled in our assembly than previously published insect genomes [9], [10], further supporting the high quality of the TGL genome (Figure S3A).

In total, 19,642 genes were annotated in *E*. *onukii* and compared with five other well-annotated Hemiptera published genomes including *Diuraphis noxia* [12], *Acyrthosiphon pisum* [13], *Cimex lectularius* [9], *Nilaparvata lugens* [14], and *Myzus persicae* (https://bipaa.genouest.org/sp/acyrthosiphon_pisum/). Results showed that 77.8% (15,273/19,642) of *E*. *onukii* genes had homologs with at least one of the five Hemiptera insects, among which 56.9% (11,170/19,642) were shared in all the six genomes examined (Figure 1B). The *E*. *onukii* genome contained ∼ 37.7% repetitive sequences, a relatively moderate level among published Hemiptera genomes, of which repetitive sequences ranged from ∼ 12% in *D*. *noxia* [12] to 56.5% in *C*. *lectularius* [9]. Transposable elements (TEs) accounted for ∼ 33.5% of the *E*. *onukii* genome, and were composed of DNA transposon (11.96%) and retroelement (21.53%) (Table S8). TE level in *E*. *onukii* was comparable to that in *N*. *lugens* [15], but approximately 1.5 times higher than that in *A*. *glycines* [10]. Similar to the aphid genome, long interspersed nuclear elements (LINEs) were more prevalent than LTR retroelements (Table S8). The moderate genome size and the levels of repetitive sequences in *E*. *onukii* compared to other hemipteran species (Figure 1C, Figure S3B) indicated that TEs and other non-coding DNA sequences might contribute to variations in genome size [16]. Consigering that the variations in genome size of insect generally result from changes in the DNA repetitive content [16], we calculated the correlation between DNA repetitive content and genome size in sequenced insect species from both the Holometabola (*e.g.*, flies, beetles, wasps, and butterflies) and Hemimetabola (*e.g.*, aphids, true bugs, blood-feeding bugs, and leafhoppers). As expected, the genome size was highly correlated with the DNA repetitive content (Spearman’s correlation, *r* = 0.8, *P* = 0.0004) (Figure S3B). Previous studies have suggested that differences in DNA repeat content are likely due to TE variation or the influence of stochastic population effects [17].

**Supplementary Figure S4:**
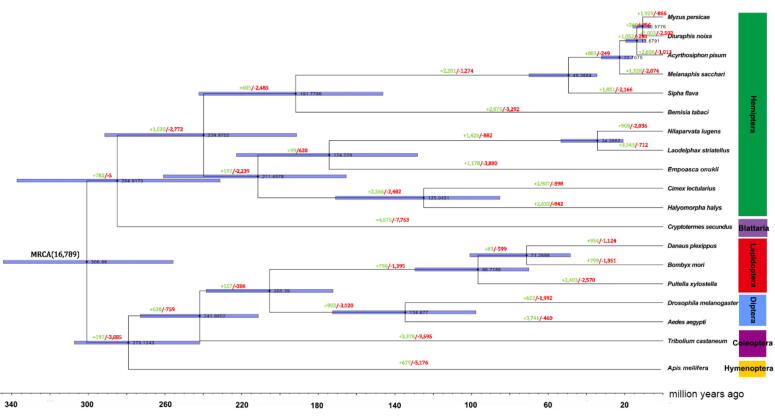
**Gene family expansions and contractions in the *E. onukii* compared with other insects** Numbers for expanded (green) and contracted (red) gene families are shown on branches.

We used OrthoFinder to identify orthologous genes across the genomes of *E*. *onukii* and other 18 insect species covering six different insect orders (Hemiptera, Isoptera, Hymenoptera, Coleoptera, Diptera, and Lepidoptera). A total of 196 single-copy orthologous genes, 6411 multi-copy orthologous genes, 18 unique paralogous genes, and 3325 unclustered genes were identified. The phylogenetic relationships among 19 sequenced insect species were analyzed using the PROTGAMMALGX model in RAxML [18] based on the 196 single-copy orthologous genes (Figure 1C). Based on these analyses, *E*. *onukii* was estimated to have diverged from *N*. *lugens* and *Laodelphax striatellus* approximately 175 million years ago (MYA) (Figure S4).

Expansion and contraction of gene families were analyzed based on 19 species. Results showed that both total and species-specific genes in Hemiptera genomes increased relative to other insect orders (Figure S4) [15]. We identified 2859 novel genes (species-specific) in *E*. *onukii*, representing about 14.5% of the genome. In addition, 1178 expanded gene families were detected and these gene families were over-represented in specific Gene Ontology (GO) terms, including carboxylic ester hydrolase activity, zinc ion binding, iron ion binding, and transmembrane transporter activity (Table S9). The *E*. *onukii* genome contained 3880 contracted gene families (Figure S4). Functional analysis revealed that these gene families were involved in immunity (immunoglobulins), myosin, and tropomyosin (Table S10). These specific gene family expansions and contractions in *E*. *onukii* were likely involved in evolutionary adaption to tea phloem sap, symbiotic dependence, pathogen immunity, environmental conditions (such as ecological and climatic variations), and tea variety differences. For example, evidence has shown that carboxylic ester hydrolase activity is involved in metabolic resistance to sequestering organophosphate (OP) insecticides in sap-sucking insects (*e.g.*, *M*. *persicae* and *Schizaphis graminum*) [19]. Immunoglobulin superfamily proteins have been reported as candidates for synapse targeting functions related to synaptic specificity in the visual system in *Drosophila* [20], [21].

### Genomic adaptation to chemoreception and detoxification

The chemosensory system is essential for herbivorous insects to orient toward and locate potential host plants [22], potentially indicating how herbivorous insects adapt to host changes. Environmental signals and chemosensory stimuli are recognized and transduced by several multi-gene families including those encoding olfactory receptors (ORs), ionotropic receptors (IRs), gustatory receptors (GRs), odorant-binding proteins (OBPs), and chemosensory proteins (CSPs) [22], [23]. To examine genes linked to chemosensory stimulus recognition, we manually annotated several related gene families, including 20 OR genes, 23 IR genes, 12 GR genes, 5 OBP genes, and 26 CSP genes (Table 3).

**Table 3:** **Number of genes encoding the proteins related to chemoreception and detoxification among different insect species**

**Insect species**	**OR**	**GR**	**IR**	**OBP**	**CSP**	**COE**	**ABC**	**GST**	**CYP**
*Acyrthosiphon pisum*	79	77	11	15	11	57	187	22	83
*Nilaparvata lugens*	50	10	25	11	17	79	40	11	67
*Empoasca onukii*	20	12	23	5	26	77	29	30	103
*Periplaneta americana*	154	522	640	6	11	90	115	39	178
*Apis mellifera*	163	10	9	21	6	24	41	11	46
*Tribolium castaneum*	299	220	23	49	20	47	73	36	131
*Anopheles gambiae*	79	76	55	82	8	40	55	28	105
*Aedes aegypti*	131	79	135	111	43	49	69	26	160
*Drosophila melanogaster*	62	68	66	51	4	35	56	38	85
*Bombyx mori*	73	76	24	44	20	76	53	20	81
*Plutella xylostella*	83	26	49	64	20	62	82	22	85

*Note*: OR, olfactory receptor; GR, gustatory receptor; IR, ionotropic receptor; OBP, odorant-binding protein; CSP, chemosensory protein; COE, carboxylesterase; ABC, ATP-binding cassette; GST, glutathione *S*-transferase; CYP, cytochrome P450.

**Figure 2:**
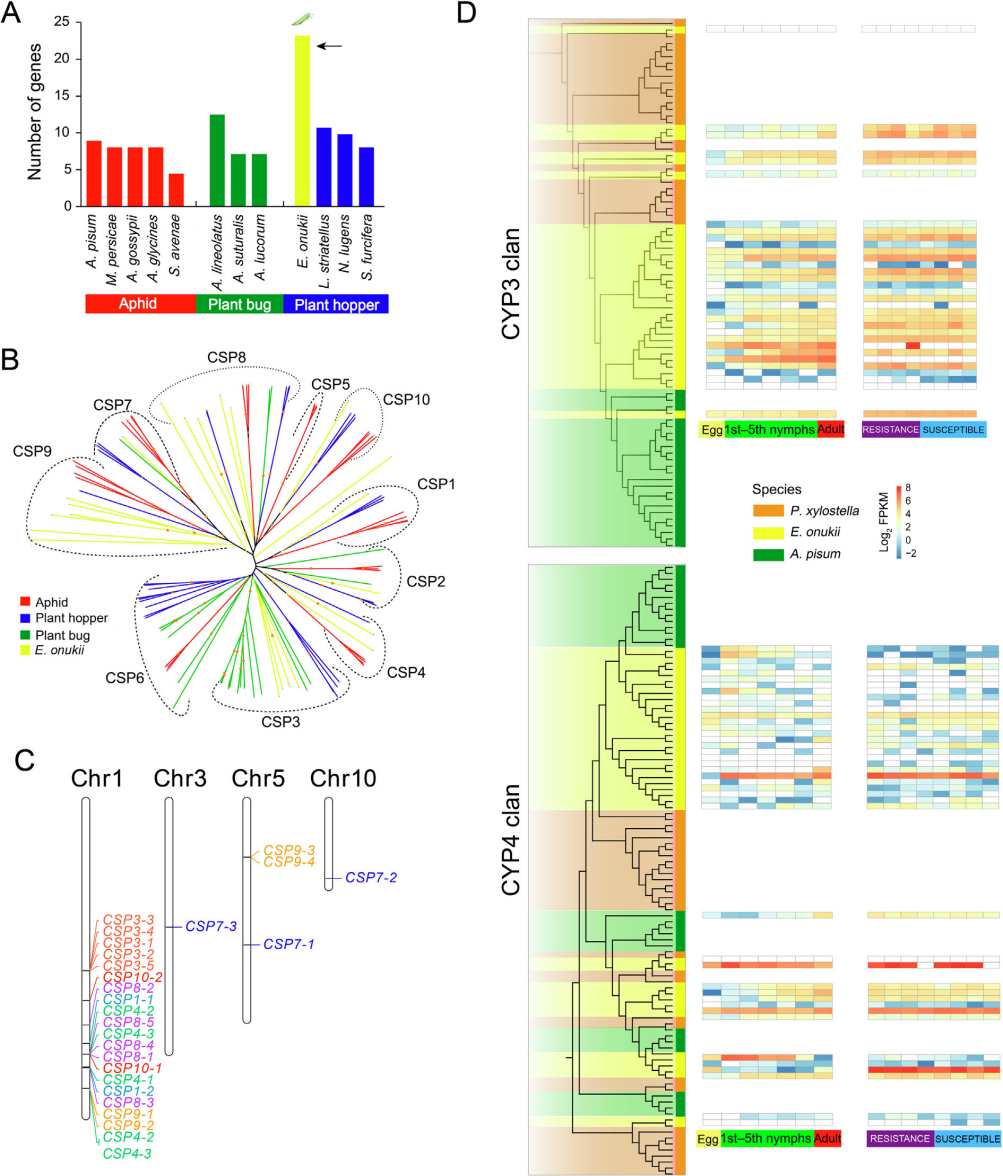
**Expansion of gene families related to chemoreception and detoxification in *E*. *onukii* compared with other insect species** **A.** Comparison of the numbers of CSP genes among aphids, plant bugs, and hoppers. Aphid species include *A*. *pisum*, *M*. *persicae*, *A*. *gossypii*, *A*. *glycines*, and *S*. *avenae*; plant bugs include *A*. *lineolatus*, *A*. *suturalis*, and *A. lucorum*; hoppers include *E*. *onukii*, *L*. *striatellus*, *N*. *lugens*, and *S*. *furcifera*. **B.** Phylogenetic relationships of CSP genes in plant hoppers (*N*. *lugens*, *S*. *furcifera*, *L*. *striatellus*, and *E*. *onukii*), aphids (*A*. *pisum*, *M*. *persicae*, *A*. *gossypii*, *A*. *glycines*, and *S*. *avenae*), and plant bugs (*N*. *lugens*, *L*. *striatellus*, and *S*. *furcifera*). Yellow branches represent CSP genes in *E*. *onukii*; blue branches represent CSP genes in *N*. *lugens*, *S*. *furcifera*, and *L*. *striatellus*; red branches represent CSP genes in *A*. *pisum*, *M*. *persicae*, *A*. *gossypii*, *A*. *glycines*, and *S*. *avenae*; green branches represent CSP genes in *N*. *lugens*, *L*. *striatellus*, and *S*. *furcifera*. **C.** Genomic expansion and unbalanced chromosomal distribution of CSP genes in the *E*. *onukii* genome*.***D.** Phylogenetic relationships and expression profiles of CYP3 and CYP4 genes in *E*. *onukii*, *A*. *pisum*, and *P. xylostella*. Expression profiles based on RNA-seq data were generated from all developmental stages (egg, 1st–5th nymphs, and adult) and 11 *E*. *onukii* populations collected from different tea cultivars (four cultivars resistant to *E*. *onukii* including LongJ, DeQ, JianD, and JuY; four cultivars susceptible to *E*. *onukii* including ZhuS, LanT, BanZ, and EnB). RNA-seq, RNA sequencing; FPKM, fragments per kilobase of transcript per million fragments mapped; CSP, chemosensory protein; CYP, cytochrome P450.

**Supplementary Figure S5:**
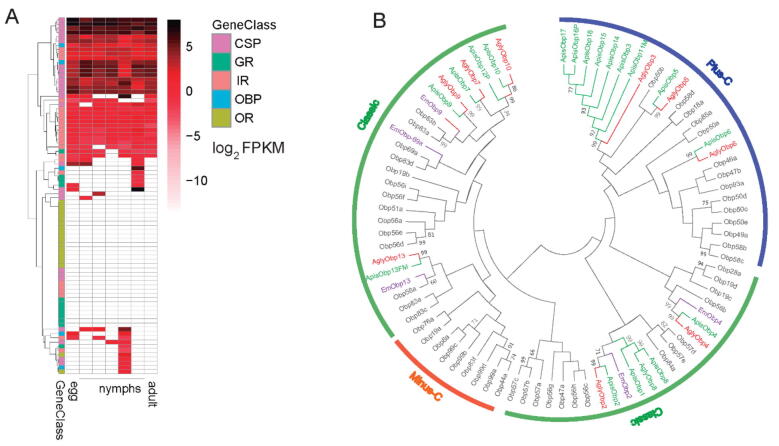
**Expression profile of genes involved in chemoreception and phylogenetic analysis of OBP gene family A.** Expression of 5 chemoreception gene families. **B.** Neighbor-joining method involving 84 OBPs was used to construct the tree. Different colors represent different species: red represents *A. glycines*; green represents *A. pisum*; purple represents *E. onukii* whereas black represents *D. melanogaster*. OR, olfactory receptor; GR, gustatory receptor; IR, ionotropic receptor; OBP, odorant-binding protein.

Comparative analysis of genomes across different species revealed an increased number of CSP genes in the *E*. *onukii* genome (Figure 2A; Table 3, Table S11). The phylogenetic analysis of Hemiptera species identified 10 homologous subgroups of CSP genes (CSP1–CSP10) (Figure 2B), which was consistent with a previous study [24]. Other than subgroups of CSP5 and CSP6, *E*. *onukii* CSP genes were present in eight of the ten clades, indicating CSP genes are highly conserved across Hemiptera. Interestingly, we found obvious expansion of some subgroups (*e.g.*, subgroups of CSP3, CSP4, CSP8, and CSP9) in *E*. *onukii* and these CSP genes were unevenly distributed over 4 of the 10 chromosomes, with enrichment on chromosome 1 (Fisher exact test, *P* < 0.00001; Figure 2C). Most CSP genes were distributed in expanded clusters on chromosomes, likely through a series of gene duplication events (Figure 2C). Meanwhile, several CSP genes were highly expressed across different life cycle stages (Figure S5A), implying their important role in the growth and development of *E*. *onukii.* Earlier studies on CSP functions [25], [26], coupled with our observations of conserved phylogeny in Hemiptera species and species-specific expansion of CSP genes, indicate that CSP genes are crucial for recognition of tea volatiles and location of potential host tea plants. These findings suggest that *E*. *onukii* may require many CSPs to specifically detect the complex molecular components of odors from different tea cultivars. Thus, our analyses highlight the directions for further experimental analysis of genes linked to host adaptation. Toward this goal, functional testing of CSP genes might identify genes that are responsible for detection of specific tea cultivars by *E*. *onukii*.

**Supplementary Figure S6:**
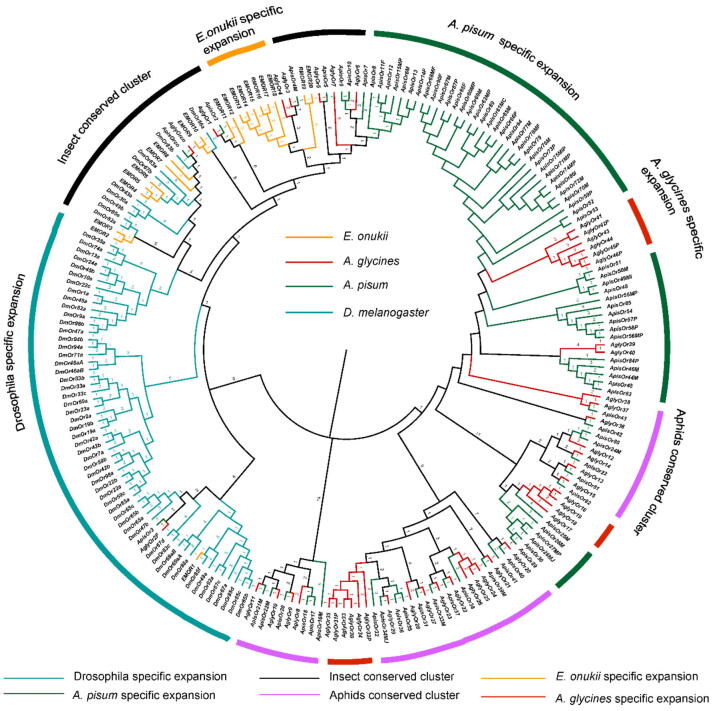
**Phylogenetic analysis of OR gene family** Neighbor-joining method involving 216 ORs was used to construct the tree. Different colors represent different species: red represents *A. glycines*; green represents *A. pisum*; yellow represents *E. onukii* whereas blue represents *D. melanogaster*.

**Supplementary Figure S7:**
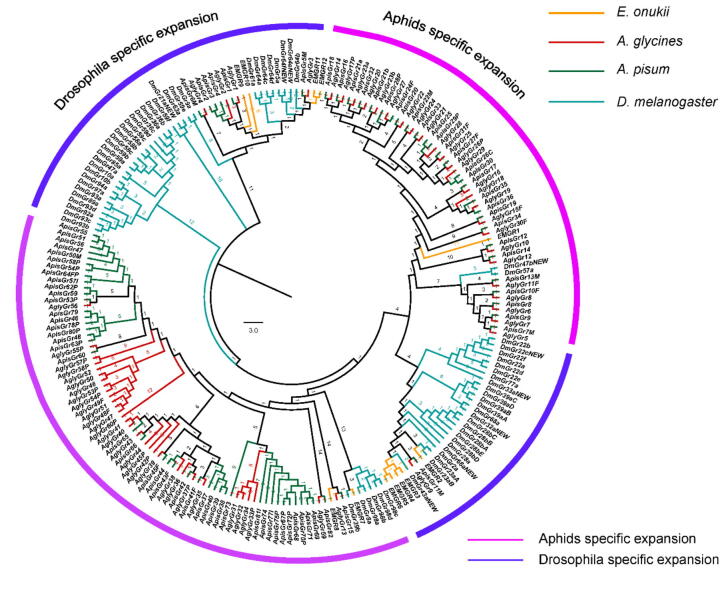
**Phylogenetic analysis of GR gene family** Neighbor-joining method involving 219 GRs was used to construct the tree. Colors corresponded to Figure S6.

**Supplementary Figure S8:**
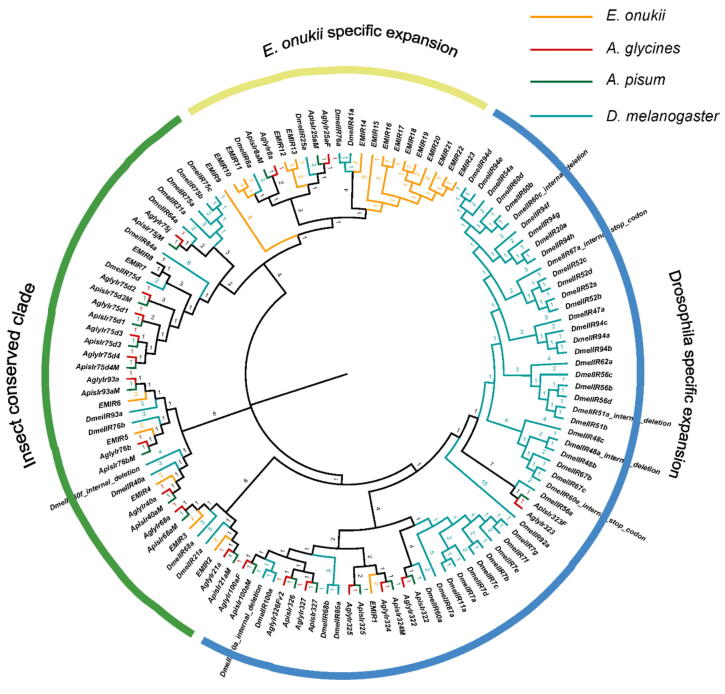
**Phylogenetic analysis of IR gene family** Neighbor-joining method involving 125 IRs was used to construct the tree. Colors corresponded to Figure S6.

Our investigation of the chemoreceptor-related genes showed relatively low number of OR genes, IR genes, GR genes, and OBP genes in *E*. *onukii* (Figures S5–S8; Table 3, Table S11). For example, we found that the number of OBP genes in Hemiptera species was lower than other insect orders (Figure S5B), suggesting conservation of odorant molecular transport in Hemiptera [15]. Other chemoreceptor-related genes, including OR genes, GR genes, and IR genes, play important roles in local adaption by responding to chemical signals with neuronal activity [15]. The species-specific expansion of OR gene family was obvious in our analysis (Figure S6). Polyphagous insects (*e.g.*, *Periplaneta americana*) possessed more OR genes than monophagous insects (*e.g.*, *E*. *onukii*) (Figure S6; Table 3, Table S11). This might have resulted from specific evolutionary adaption to food selection and detection since genetic diversity of OR genes allows insects to bind to a greater range of ligands [27]. In addition, similar to *N*. *lugens*, *E*. *onukii* had a substantially lower number of GR genes (Figure S7; Table 3). Earlier studies have shown a close relationship between GRs and insect herbivory, with a lower number of GRs in specialists than in generalists [28], [29]. Another explanation may be that antennae of leafhoppers have a much simpler structure with fewer sensilla than those of plant hoppers (*e.g.*, *N*. *lugens*) and aphids. *E*. *onukii* also possessed fewer IR genes (Figure S8; Table 3, Table S11), which mediate synaptic communication in insects and mediate responses to volatile chemicals in *Drosophila melanogaster* [30], [31]. We believed that the reduction in the numbers of OR genes, IR genes, GR genes, and OBP genes might be associated with the adaptive evolution to a monophagous diet of tea phloem sap, and the substantial expansion of CSP genes might contribute to tea volatile perception in *E*. *onukii*.

**Supplementary Figure S9:**
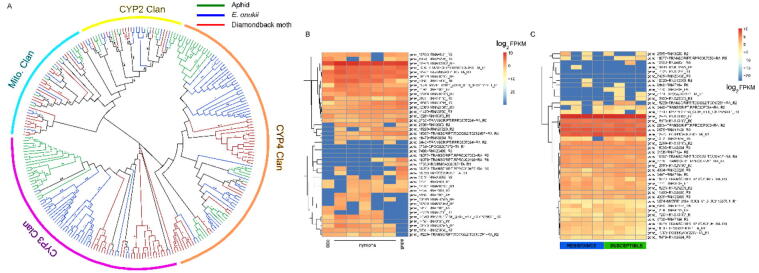
**Phylogenetic analysis of CYP gene family A.** Neighbor-joining method involving 259 CYPs was used to construct the tree. Colors represented different species listed in the Figure 2. **B.** Expression patterns of CYPs among developmental stages; **C.** Expression patterns of CYPs when living on resistant and susceptible tea cultivars.

*E*. *onukii* is believed to have experienced rapid evolution leading to insecticide resistance in natural populations [32]. Four classic gene families commonly associated with detoxification of xenobiotics and insecticides, including those encoding cytochrome P450s (CYPs), carboxylesterases (COEs), glutathione *S*-transferases (GSTs), and ATP-binding cassette (ABC) transporters, were therefore investigated. We identified 103 CYP genes, 29 ABC transporter genes, 77 COE genes, and 30 GST genes (Table 3). Similar to other insecticide resistant pests [33], we found that the CYP gene family was expanded, mainly in CYP3 and CYP4 clans (Figure 2D, Figure S9A; Table 3, Table S12). Based on our RNA sequencing (RNA-seq) data, 28 CYP3 genes and 38 CYP4 genes were expressed in *E*. *onukii* [fragments per kilobase of transcript per million fragments mapped (FPKM) > 1.0], with 20 CYP3 genes and 16 CYP4 genes being highly expressed during at least one developmental stage (FPKM > 10.0) (Figure 2D, Figure S9B). The results underlined their potential function of detoxifying the xenobiotics or insecticides in *E*. *onukii*.

We tracked the expression patterns of these genes in 11 *E*. *onukii*  samples collected from different tea cultivars, with four cultivars resistant and four cultivars susceptible to *E*. *onukii*  according to previous studies [34]. Results showed that 17 CYP3 genes and 12 CYP4 genes were highly expressed (FPKM > 10.0) but not differentiated in both resistant and susceptible tea cultivars (Figure 2D; Figure S9C). Thus, we speculated that CYP genes might be involved in metabolism of common xenobiotics, or their expression may be induced by insecticides. Indeed, previous studies have shown that CYP3 genes are involved in xenobiotic metabolism and insecticide resistance, with some family members being inducible by pesticides or plant secondary metabolites [35]. CYP4 genes are known to encode constitutive and inducible enzymes related to odorant and pheromone metabolism, and their expression can be induced by xenobiotics [36].

### Niches under adaptive selection are related to metabolic regulation and detoxification

**Figure 3:**
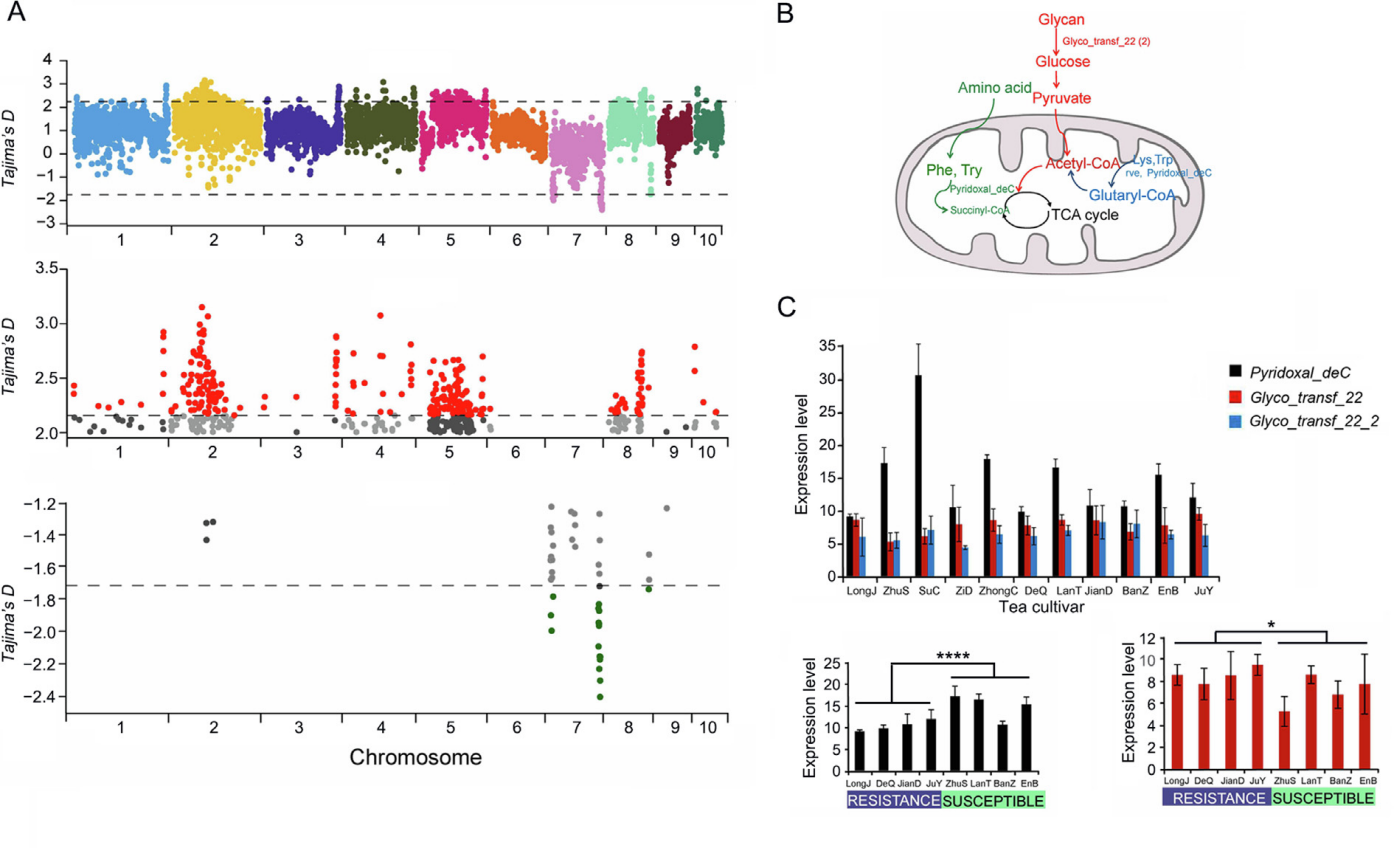
**Genomic signatures of balancing selection** **A.** Putative selection sweeps in populations of *E*. *onukii*. Tajima’s *D* value was calculated for each of the *E*. *onukii* populations. Mean values of Tajima’s *D* are shown in sliding windows of 50 kb with a step size of 10 kb. Regions with Tajima’s *D* values significantly deviating from 0 are marked with dotted lines in upper panel. Specifically, regions with Tajima’s *D* values significantly deviating from 0 are further plotted in red (> 2.15753) and green (< −1.72491) in middle and lower panels, respectively. **B.** Succinylation and glutarylation pathways showing the regulatory role of lysine modifications in metabolism. **C.** Expression patterns of the *E*. *onukii* genes under balancing selection in 11 different tea cultivars. *Pyridoxal_deC* was detected to be significantly highly expressed in susceptible tea cultivars (****, *P* < 0.0001; Student’s *t*-test), while *Glyco_transf_22* was significantly highly expressed in resistant tea cultivars (*, *P* < 0.05; Student’s *t*-test). CoA, coenzyme A; TCA, trichloroacetic acid.

*E*. *onukii* samples were collected from four tea-growing regions in China: southwestern region (SWR), south of the Yangtze River region (SYR), north of the Yangtze River region (NYR), and southeastern region (SER), and these samples were resequenced with a depth ranging from 20.5× to 34.7× at whole-genome level (Table S13). After filtering the low-quality variants, we generated a genomic dataset containing 12,271,501 high-quality SNPs (Table S13) to estimate the genomic signatures of evolutionary adaptation for *E*. *onukii*, based on Tajima’s *D* with a 50-kb window size and a fixed step length of 10 kb. Totally 369 sliding windows, covering 18.45-Mb genomic sequences (Table S14) and containing 86 protein-coding genes (7 under purifying selection and 79 under balancing selection; Figure 3A; Tables S15 and S16), were detected.

Purifying selection is important in shaping genomic diversity in natural populations and is essential to preserving biological functions at selection sites [37]. Almost all the seven genes were related to nervous system or visual functions. For instance, forkhead box protein P1 (*FOXP1*) encodes a transcription factor with a regulatory function in the central nervous system (CNS), and mutations in this gene have been linked to various neurodevelopmental diseases, including autism, cognitive abnormalities, intellectual disabilities, and speech defects [38]. Coronin 6 (*CORO6*) encodes a protein which is highly enriched at the adult neuromuscular junction and can regulate acetylcholine receptor (neurotransmitter receptor) clusters by modulating interactions between the actin cytoskeletal network and receptors [39]. In mice lacking seizure threshold 2 (*SZT2*), mechanistic target of rapamycin (mTOR) complex 1 (mTORC1) signaling is hyperactive in several tissues including neurons in the brain, and these components have been linked to neurological diseases [17]. The nucleoredoxin-like 1 (*Nxnl1*) gene has two alternative splice isoforms, one of which encodes a rod-derived cone viability factor that functions in the retina [40]. In mammal, diphosphoinositol pentakisphosphate (IP7) is produced by a family of inositol hexakisphosphate kinases, which have distinct biological functions including energy homeostasis associated with neuroprotection [41]. In insects, detections of light changes, vibration, colors, and semiochemicals, which have evolutionarily old sensory functions, are vital for behaviors including avoiding predation, food location, and intraspecific communication. Thus, we speculated that the genes under purifying selection would be important for nervous or visual functions in *E*. *onukii*.

**Supplementary Figure S10:**
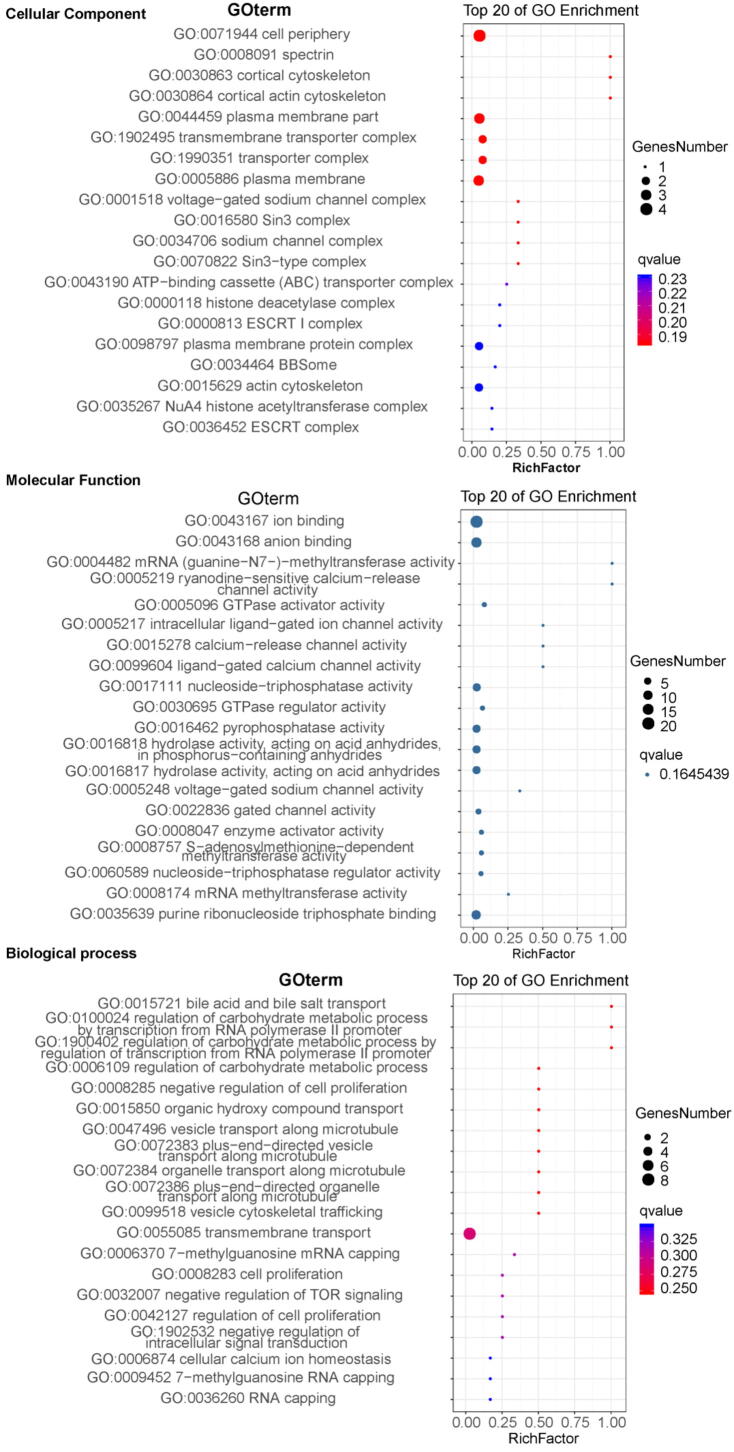
**GO enrichment of the genes under balancing selection in *E. onukii*** Only top 20 are listed in the figure. GO, Gene Ontology.

We found that 91.8% of the selected genes (79/86) were under balancing selection with positive Tajima’s *D* values that greatly deviated from zero, indicating that the *E*. *onukii* populations maintained a high level of polymorphism. The average pairwise diversity index (*π*) were calculated to evaluate the genetic diversity. A much higher genetic diversity (*π* = 0.00804) was observed in those genomic regions under balancing selection compared to genome-wide diversity (*P* < 0.0001, Student’s *t*-test), suggesting a strong capacity for *E*. *onukii* to rapidly adapt to diverse habitats [42]. GO enrichment analysis showed that these genes were enriched in several biological processes including cell periphery, plasma membrane part, transmembrane transport, ion binding, anion binding, and nucleoside-triphosphatase activity (Figure S10; Table S17). Kyoto Encyclopedia of Genes and Genomes (KEGG) enrichment analyses pointed to several pathways including those linked to metabolism, circadian rhythms, and immune system functions.

**Supplementary Figure S11:**
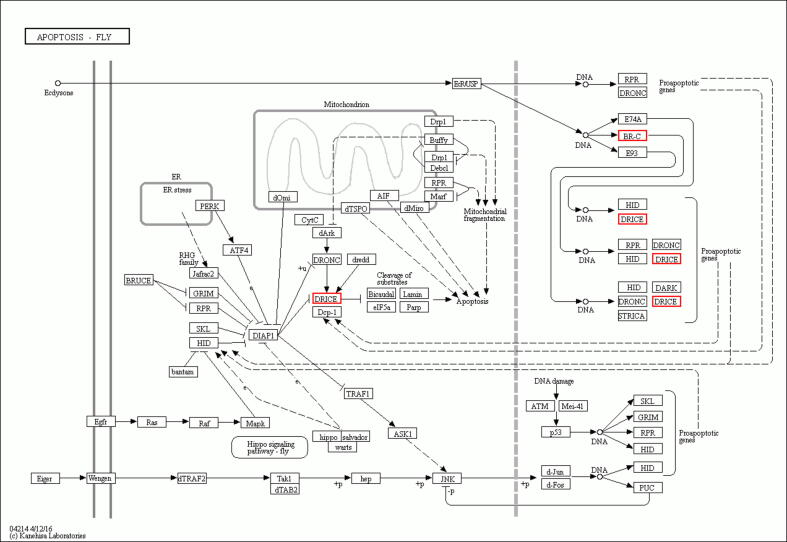
**Apoptosis pathway was enriched for the genes under balancing selection in *E. onukii*** Genes under selection are marked in red.

Based on KEGG analyses, lysine succinylation and glutarylation pathways were enriched (Figure 3B). Previous studies have reported that protein acetylation plays critical roles in cellular processes ranging from gene expression to metabolism [43]. Lysine succinylation is a recently identified post-translational modification (PTM) [44]. It is important for metabolism and detoxification in *Bombyx mori* [44]. In our study, the apoptosis pathway was consistently enriched (Figure S11). Studies in Lepidoptera insects suggest that apoptosis plays a vital role in resistance to virus infection and some apoptosis-related proteins are known to be succinylated [39], [45], [46]. Here, we identified four key genes, including *rve*, *Pyridoxal_deC*, *Glyco_transf_22*, and *Glyco_transf_22_2*, which were functionally involved in lysine succinylation and glutarylation pathways (Figure 3B).

Lysine succinylation is important for virus-infection resistance and detoxification in insects [43], [44], [45], [47], [48]. To examine whether the selected pathways in *E*. *onukii* have similar functions, we analyzed the expression patterns of four genes including *rve*, *Pyridoxal_deC*, *Glyco_transf_22*, and *Glyco_transf_22_2*. Based on RNA-seq analysis, *Pyridoxal_deC* showed significantly high expression in susceptible tea cultivars (*P* < 0.01, Student’s *t*-test; Figure 3C), while *Glyco_transf_22* showed significantly high expression in resistant tea cultivars (*P* < 0.05, Student’s *t*-test; Figure 3C). *Pyridoxal_deC* and *Glyco_transf_22* were key genes in metabolic regulation of succinyl-CoAs and glutaryl-CoAs (Figure 3B). These results, together with the previously reported roles of succinylation and glutarylation in other insects [43], [44], [45], [48], indicate that genes under balancing selection could be involved in metabolic regulation and detoxification of *E*. *onukii*, possibly contributing to its success in adapting to a wide range of tea cultivars grown in ecologically diverse regions of China.

**Supplementary Figure S12:**
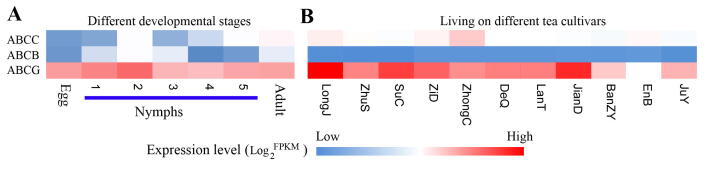
**Expression patterns of the ABCs under balancing selection A.** Expression patterns of ABCs among developmental stages; **B.** Expression patterns when living on resistant and susceptible tea cultivars. ABC, ATP-binding cassette transporter.

*E*. *onukii* is largely controlled using insecticides in China, leading to development of resistance to chemicals. ABC transporters are conserved across insects and have been implicated in insecticide resistance among pest species [49]. Based on our analyses, the ABC superfamily showed no expansion, and few orthologs were present in *E*. *onukii* compared to other insect species (Table 3). However, we identified three ABC transporter genes that showed signatures of balancing selection and maintained high genetic variations within populations of *E*. *onukii*. Further analysis showed that these three genes belonged to three ABC subfamilies including ABCG (gene_12883-RNA1987_R0), ABCB (gene_2789-MYZPE13164_G006_V1.0_000121650.3_R0), and ABCC (gene_16942-RNA135_R0). Balancing selection favors defense proteins with functions in resistance, immunity, and adaptations [50], [51]. However, the functions of these three genes have not been elucidated in leafhoppers or aphids. Studies in other animals or insects have shown that these subfamilies are closely related to drug or insecticide resistance [52], [53], [54]. A comparative analysis between susceptible and resistant strains of *Aedes aegypti* reports that the genes of ABCG family are highly up-regulated in the resistant strains [53]. Similar studies have also been carried out in *Plutella xylostella* and *L*. *striatellus*, showing that ABCB/G subfamilies are significantly overexpressed in the resistant strains [54], [55]. Based on ABC family functions in other insects, we hypothesized that *E*. *onukii* ABC transporter genes might contribute to its adaptation to different tea cultivars. This hypothesis may be supported by a study of Cry1Ac resistance in *P*. *xylostella* [52]*.* We therefore investigated the expression patterns of the ABC transporter genes that we identified (ABCB, ABCC, and ABCG) using *E*. *onukii* samples collected from 11 different tea cultivars, as described above. These genes showed moderate expression levels across different developmental stages (Figure S12A) and samples of *E*. *onukii* from different tea cultivars (Figure S12B), suggesting that these ABC superfamily members could broadly contribute to adaptation to various tea cultivars and even possibly to chemical resistance. Previous studies also suggest that ABC transporters are not strictly specific to certain chemicals, implying that ABC transporters have a broad spectrum of chemical substrates and may act as a basis for cross-resistance of multiple chemicals [54].

Genomic regions under balancing selection are functionally important because of their high genetic diversity contributing to adaption to environmental changes [42]. Based on our results, we hypothesized that balancing selection might have contributed to the high-level polymorphism in *E*. *onukii* populations, facilitating adaptation to diverse environments and tea cultivars.

### Evolutionary history is inconsistent between TGL and tea

**Figure 4:**
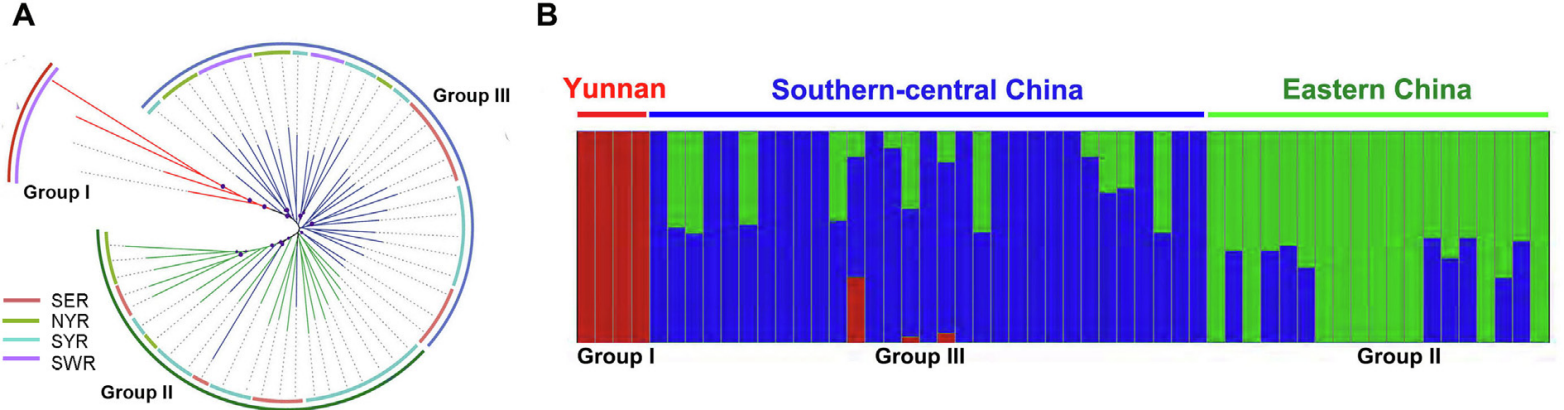
**Phylogenetic relationship, population structure,and expansion of *E*. *onukii*** **A.** Phylogenetic tree of the 54 *E*. *onukii* samples based on RAxML and SplitsTress. Branch lengths are not scaled. Different colors of inner circle represent 4 different tea-growing regions in China. Colors of outer lane represent different *E*. *onukii* groups based on phylogenetic analysis. **B.** Genetic structure and individual ancestry with colors in each column representing ancestry proportion over range of population sizes (*K* = 2–4, with an optimal *K* = 3). SWR, southwestern region; SYR, south of the Yangtze River region; NYR, north of the Yangtze River region; SER, southeastern region.

**Supplementary Figure S13:**
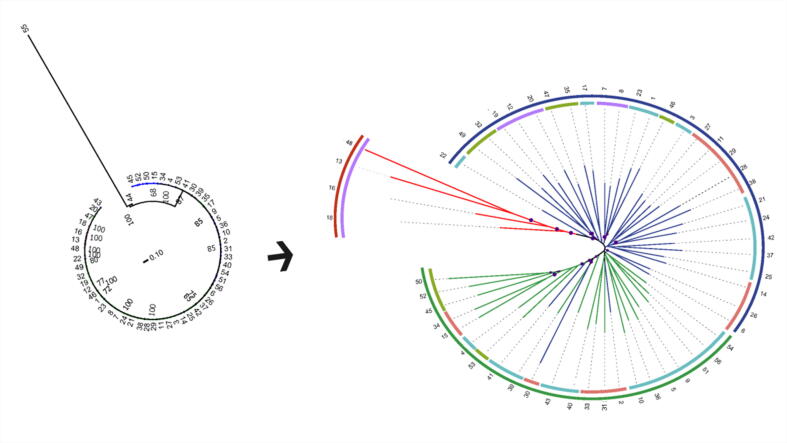
**Phylogenetic tree and network estimation by RAxML and SplitsTress** Three populations according to the geographic regions that most of the individuals located: Yunan (group I), Eastern China (group II), and central and southern of China (group III) are listed in red, green, and blue, respectively, (right figure) with presence of the outgroups (left figure). Different 4 tea regions of China are marked in different colors represented in Figure 4. Details of the IDs in the phylogenetic tree are listed in Table S13.

**Supplementary Figure S14:**
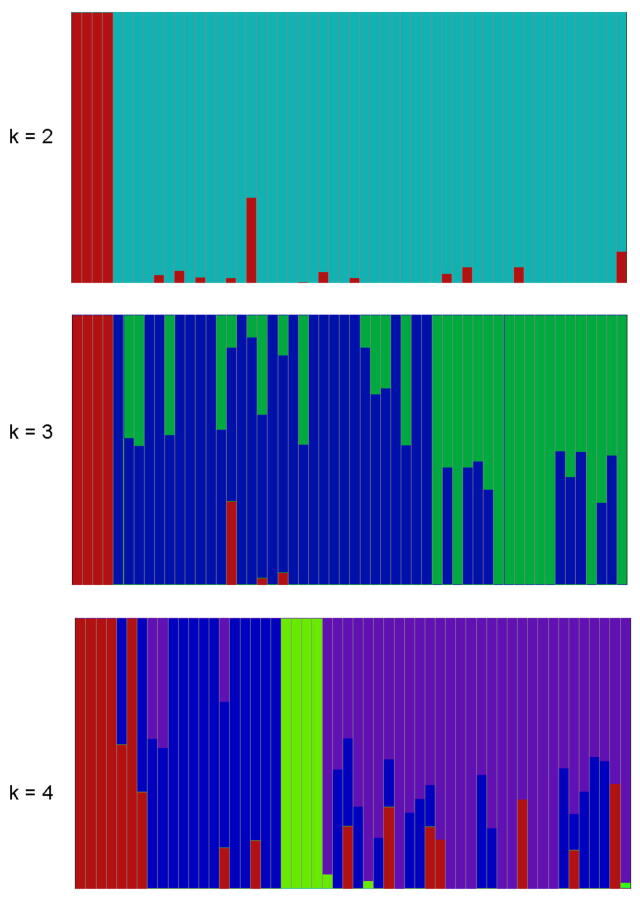
**Admixture analysis of 55 TGLs accessions (*K* = 2–4)**

We used high-quality SNPs obtained from the 54 *E*. *onukii* samples coming from different tea-growing regions in China (Table S13) to profile their phylogeographical relationships. Phylogenetic analysis and network estimation of the *E*. *onukii* samples with *Empoasca flavescens* and *Asymmetrasca* sp. as outgroups uncovered three geographically clustered groups (Groups I–III; Figure 4A, Figure S13). Group I contained 4 samples collected from Yunnan province, being the closest to the outgroups. Group II included 28 samples mainly collected from eastern China, including Shandong, Jiangsu, Zhejiang, and Anhui provinces. The remaining 22 samples (Group III) were mainly from 13 provinces in central and southern China (Figure 4A; Table 4**)**. These results were further supported by genetic structure analysis (*K* = 3) based on the Admixture model (Figure 4B, Figure S14) [56]. Three clustered groups of *E*. *onukii* samples (Figure 4; Table 4) were inconsistent with the current division of the four tea-growing regions based on the tea growing history, geographical locations, and tea cultivars [57]. These results suggest a different evolutionary history of *E*. *onukii* among these regions. Our analyses of phylogenetic and genetic structure confirmed the genetic differences between Group I (samples from Yunnan province) and the other two groups, as reported in a previous study based on microsatellites [5]. However, this previous study suggested four main genetic groups (*K* = 4) [5]. This may be because the present study collected much more samples in China (54 locations in 22 provinces) than the other one (19 locations in 13 provinces) and used a greater number of genetic markers (whole-genome SNPs *vs.* microsatellite markers). We observed that individuals from different groups were interspersed (Figure 4), possibly reflecting gene flow across location, as observed in the previous study [5].

**Table 4:** **Number of populations, nuclear SNPs, andInDels, as well asgenetic diversity in each of the three clustered groups**

**Group**	**No.ofpopulations**	**No.ofSNPs**	**No.ofInDels**	**Genetic diversity** (***π*)**
Group I	4	52,089,001	14,913,280	0.004062
Group II	28	472,824,888	135,096,045	0.004744
Group III	22	279,181,783	79,642,551	0.004662

*Note*: SNP, single nucleotide polymorphism; InDel, insertion and deletion.

To investigate the genetic divergence, we calculated *π* within each of the clustered groups. Comparably higher levels of genetic diversity were observed in Group II and Group III than in Group I (*P* < 0.001; Table 4). The high genetic diversity of *E*. *onukii* samples in eastern China and southern-central China may be explained by a geographically wide range and ecologically diverse tea-growing conditions. Further, we found a higher genetic diversity in certain subgroups within the major tea-growing provinces in eastern China and southern-central China. The diversity (*π* = 0.004801) in Jiangsu and Zhejiang provinces was higher than the overall diversity in eastern China (*π* = 0.004744). Similarly, a higher diversity (*π* = 0.004939) was found in Anhui and Fujian provinces than the overall diversity in southern-central China. We further analyzed the population genetic differentiation (*F*_ST_) across different geographically clustered groups, and found a very low *F*_ST_ value (0.005902) between Group II and Group III, indicating their genetically close relationship. In contrast, the *F*_ST_ value between Group I and Group II or Group III was much higher (*F*_ST_ = 0.052321 in Group II *vs.* Group I; *F*_ST_ = 0.043225 in Group III *vs.* Group I), suggesting that the samples from Yunnan province were genetically distant from the populations in eastern China and southern-central China.

A previous study has reported that the structure of male genitalia varies among *E*. *Onukii* populations from eastern China, southern-central China, and Yunnan province [3]*.* Our population genetic analysis also showed genetic differences among these different groups (Figure 4). We speculated that some geographical barriers might have restricted gene flow leading to these differences. Yunnan is surrounded by mountains and rivers as a result of an uplift during the Quaternary and is isolated by the steep Hengduan Mountains. Unlike the clonal propagation of tea cultivars in other regions of China, tea cultivation in Yunnan has depended on seeds from early times [58]. This might have prevented the interbreeding of local *E*. *onukii* populations with populations from other regions. Similarly, the hilly region between Zhejiang and Fujian might separate *E*. *onukii* populations, leading to populations genetically different (Figure 4).

## Conclusion

In this study, we generate a high-quality chromosome-level genome with 92.7% BUSCO completeness for *E*. *onukii*, a species of crucial importance to a widely consumed crop linked to human health. Based on genomic profiling and comparison, we find complex patterns of genomic variation and expansion of gene families associated with evolutionary adaptation to chemosensory reception and xenobiotic detoxification. We identify genomic signatures of balancing selection to reveal the high genetic diversity of resistant genes, underlining their important roles in the adaptive evolution of *E*. *onukii*. Further, we analyze patterns of variation in genomic sequences from 54 samples and two outgroups, uncovering the population structure and evolutionary history of *E*. *onukii* across the four different tea-growing regions of China. This work will facilitate functional studies on the adaption of this pest to ecologically diverse habitats, and provide the genomic resources and genetic knowledge for development of sustainable pest management strategy.

## Materials and methods

### Insect colony

*E. onukii* samples were collected in Fuzhou, Fujian Province, in the southeast of China in July 2017 (on the tea cultivar of Huangdan), and then maintained on tea plants in the laboratory. The insectarium environment was set at 28°C ± 1°C and 60% ± 5% relative humidity (RH) with a 12 h:12 h light-dark photoperiod.

### Genome sequencing and assembly

Since the quality of *de novo* assembly is sensitive to genomic heterozygosity, genomic DNA of adults was extracted from insects after 12 generations of laboratory inbreeding (details in File S1). Chromosome-level assembly was performed using ONT with Hi-C (File S1). The raw ONT reads were self-corrected using Canu (v1.7) [60] with parameter corOutCoverage = 100, and corrected reads were subject to two widely used long-read assemblers, wtdbg2 [61] and SMARTdenovo (https://github.com/ruanjue/smartdenovo). These two assemblers applied the homopolyer-compressed (HPC) *k*-mer indexing algorithm for sequence alignment and assembly, making the heterozygous regions prone to collapsing. To improve the contiguity of contig assemblies, we used Quickmerge [62] to reconcile wtdbg2 and SMARTdenovo assemblies. Each round of assemblies was inspected through evaluation of N50s based on assembled genome size as well as complete/duplicated BUSCO ratio (Table S3), showing 92.7% completeness and only 2.7% duplication. It also indicated that the redundant sequences were well-handled in our assembly. The total length of the final contig assembly for *E*. *onukii* genome was 599 Mb with a contig N50 size of 2.2 Mb. Illumina short reads were then used to polish the ONT-assembled genome by Pilon [59] with the following parameters: --diploid --threads 6 --changes --tracks --fix bases --verbose --mindepth 4. Hi-C libraries were created from nymphs as previously described [33]. The original cross-linked fragments, also known as chimeric fragments, were then processed into paired-end sequencing libraries and sequenced on the Illumina HiSeq X10 platform. Paired-end reads were uniquely mapped onto the draft assembly, and then 3D-DNA pipeline [63] was recruited to correct any mis-joined contigs by detecting abrupt long-range contact patterns. The Hi-C corrected contigs were further linked into 10 pseudo-chromosomes using the ALLHiC pipeline [64]. In total, we generated 65 Gb sequence data (∼ 109×) for one cell by ONT and 37 Gb clean data (∼ 61×) of Illumina HiSeq X10 from polishing (Table S1).

### Official gene set annotation

Annotation of protein-coding genes was based on *ab initio* gene predictions, transcript evidence, and homologous protein evidence, which were all implemented in the GeMoMa computational pipeline [65]. RNA-seq data were generated from every developmental stage (egg, 1st–5th nymphs, and adult). Besides, multiple studies have shown that resistance to *E*. *onukii* varies with different tea cultivars [34], [66]. *E*. *onukii* samples were collected from 11 main tea cultivars in China, of which four were susceptible to *E*. *onukii* (ZhuS, LanT, BanZ, and EnB), four were resistant (LongJ, DeQ, JianD, and JuY), and three had unknown resistance status (SuC, ZiD, and ZhongC) [34]. RNA-seq reads were first trimmed using the Trimmomatic program [67] and then mapped to the reference genome using HISAT2 [68]. During homology-based prediction, the protein sequences of *D*. *melanogaster*, *Apis mellifera*, *M*. *persicae*, *A*. *pisum*, *Tribolium castaneum*, and *B*. *mori* were downloaded and aligned to the reference assembly using TBLASTN (E-value < 1E−5), and the resulting alignment files were subject to GeMoMa annotation.

### Orthology and phylogenomics

A total of 19 representative insect species including *E*. *onukii* were collected for orthology and phylogenetic analyses (Figure 1C). A phylogenetic tree based on a concatenated sequence alignment of the single-copy gene families from *E*. *onukii* and other insect species was constructed. We identified 5736 single-copy genes in these insect genomes using OrthoFinder (v2.0.0) [69] and performed multiple alignments of the single-copy genes from the selected genomes using MAFFT (v7.299b) [70]. Based on a concatenated sequence alignment, a phylogenetic tree was constructed using RAxML software and the PROTGAMMALGX model [18]. Divergence time of the selected insect species was calculated by PAML (v4.8a) mcmctree [71]. The Markov chain Monte Carlo (MCMC) was run for 1,000,000 iterations using a sample frequency of 100 after a burn-in of 2000 iterations, with the other parameters set as defaults. The following constraint was used for time calibrations: the divergence time of *D*. *melanogaster* and *A*. *mellifera* was around 42.8–83.4 MYA [72]. FigTree (v1.44) was used to visualize the phylogenetic tree. Gene family expansion and contraction analyses were performed using CAFE (v4.0.1) [73].

### Identification and analysis of gene families

Some gene families with functional importance were selected for manual annotation based on the high-quality assembly. Most gene families were annotated using known models from previously annotated genomes including *D*. *melanogaster*, *A*. *mellifera*, *M*. *persicae*, *A*. *pisum*, *C*. *lectularius*, and *B*. *mori*. Some gene families, which were difficult to identify from automated predictions, were identified based on iterative searching. In brief, BLASTP searches for Hemiptera homologs used queries to search the genomic loci for significant hits (E-value < 1 × 10^−^^3^). Further, we recruited hidden Markov models (HMMs) to identify certain domains for these selected gene families based on pfam_scan [74]. Multiple sequence alignments of the selected gene families were obtained with MUSCLE [75] and corrected manually. Phylogenetic analysis was conducted using maximum likelihood (ML) and neighbor-joining (NJ) algorithms implemented in MEGA7 for 500 bootstraps [76].

### Differential gene expression

RNA-seq data were generated from seven developmental stages (egg, 1st–5th nymphs, and adult) and 11 populations of *E*. *onukii* collected from different tea cultivars (described in “Official gene set annotation” section). The RNA-seq reads were trimmed using the Trimmomatic program [67] and mapped back to gene models using bowtie [77]. FPKM was calculated based on the RSEM program [78] implanted in Trinity software [79]. Significantly differentially expressed genes were detected with a cutoff of *P* < 0.05 and |log_2_ fold change| > 1 [80].

### Sample collection, resequencing, and SNP calling

Individuals of *E*. *onukii* (100–120) were collected from 54 plantations distributed in four tea-growing regions of China including SWR, SYR, NYR, and SER (Table S13). We also collected two samples, *E*. *flavescens* (collected from Canada in vineyards) and *Asymmetrasca* sp. (collected from Africa), as outgroups (Table S13). For DNA extraction, 50–100 individuals were mixed. Genomic DNA extraction, library construction, and amplification were performed following standard protocols (File S1). All samples were sequenced using the Illumina HiSeq X10 platform with a paired-end read length of 150 bp. The GATK (v3.5-0-g36282e4) [81] and SAMtools/BCFtools [82] were used to detect variants and SNPs following a series of filtering steps as detailed in File S1.

### ML tree inference

The phylogenetic tree was built based on SNPs of single-copy genes. The heterozygous and homozygous SNPs were included in the construction of ML tree. For the heterozygous SNPs, the major alleles that had more reads supported than the minor alleles were retained for further analysis. These SNPs were converted to phylip format and aligned in fasta format. The ML tree was constructed using IQ-Tree with a self-estimated best substitution model [83].

### Admixture analysis

Ancestral population stratification among the resequenced *E*. *onukii* populations was inferred using Admixture software [84]. We estimated the optimal ancestral population structure using ancestral population sizes of *K* = 1–4 and estimated parameter standard errors based on bootstrapping of 2000.

### Diversity statistics

VCFtools (v0.1.3) [85] was used to calculate population diversity statistics. *F*_ST_ and *π* were estimated based on a sliding window analysis with 100-kb window size and 50-kb step size.

### Scanning loci under selective sweeps

To identify candidate genes responsible for reciprocal selection in the *E*. *onukii* populations, we performed Tajima’s *D* test to identify selective sweeps. Locus with Tajima’s *D* that greatly deviated from 0 was proved to be a selection niche in the genome. Tajima’s *D* statistics were calculated using VCFtools program with 50-kb window size and 10-kb step size. A negative Tajima’s *D* indicates population size expansion and/or purifying selection. A significantly positive Tajima’s *D* signifies low levels of low- and high-frequency polymorphisms, indicating a decrease in population size and/or balancing selection [86]. We used the empirical 5% windows to indicate the significance. The lowest 5% windows were considered as purifying selection and the highest 5% windows were considered as balancing selection.

Based on the annotation of our high-quality genome, candidate genes were identified using our outliers. GO annotation was conducted using Blast2GO [87] and the KEGG pathway analysis was performed using OmicShare tools (https://www.omicshare.com/tools).

## Data availability

The genome sequences and resequencing reads have been deposited in BioProject at the National Center for Biotechnology Information (BioProject: PRJNA731240), which are publicly accessible at https://www.ncbi.nlm.nih.gov/bioproject. Reads for RNA-seq have been deposited in the Genome Sequence Archive [88] at the National Genomics Data Center (NGDC), Beijing Institute of Genomics (BIG), Chinese Academy of Sciences (CAS) / China National Center for Bioinformation (CNCB) (GSA: CRA005085), and are publicly accessible at https://ngdc.cncb.ac.cn/gsa. The mitochondrial sequences have also been deposited in the Genome Warehouse [89] at the NGDC, BIG, CAS / CNCB (GWH: GWHBAZN00000000), and are publicly accessible at https://ngdc.cncb.ac.cn/gwh.

## Competing interests

The authors declare that they have no competing interests.

## Supplementary material

Supplementary File S1 **The genome assembly pipeline**

Supplementary Table S1 **Statistics of genomic sequencing data of *E. onukii***

Supplementary Table S2 **Statistics of Hi-C mapping**

Supplementary Table S3 **Statistics of contig level assembly of *E. onukii***

Supplementary Table S4 **BUSCO analysis of genome assembly of *E. onukii***

Supplementary Table S5 **BUSCO analysis of annotation completeness**

Supplementary Table S6 **The statistics of different Hemiptera species assemblies**

Supplementary Table S7 **Assessment of genome consistency based on NGS (Illumina) reads**

Supplementary Table S8 **Statistics of TEs in *E. onukii* genome**

Supplementary Table S9 **GO over-representation of gene families expanded on *E. onukii* branch**

Supplementary Table S10 **Gene family contraction analysis on *E. onukii* branch**

Supplementary Table S11 **Chemosensory related gene families in *E. onukii***

Supplementary Table S12 **List of gene families involved in detoxification**

Supplementary Table S13 **Geographic distributions and population structure of the collected samples around China**

Supplementary Table S14 **Genomic regions under selection**

Supplementary Table S15 **Genes under selection**

Supplementary Table S16 **Functional analysis of genes under purifying selection**

Supplementary Table S17 **GO terms for the genes under balancing selection**
