# Supplementary material for: Genomic Variations in the Tea Leafhopper Reveal the Basis of Its Adaptive Evolution
Source: Genomics Proteomics Bioinformatics. 2022 Aug 28;20(6):1092–105. doi: 10.1016/j.gpb.2022.05.011 (PMC10225489; doi:10.1016/j.gpb.2022.05.011)
Supplement: Supplementary File S1 — The genome assembly pipeline [file mmc1.docx]

**File S1 The genome assembly pipeline**

**Insect samples**

Insects were collected in the South Mountain located in Fujian Agriculture and Forest University and maintained in the lab by inbreeding for 12 generations. The adults were collected for further analyzed.

**Illumina short reads sequencing**

DNA was extracted using TIANamp Genomics DNA Kit (dp304, TIANGEN, Beijing, China) and sequenced on Illumina HiSeq X10 platform with 150-bp reads length and insert size of 300–500 bp (Table S1).

**Genome Size and heterozygosity estimation**

Genome DNA was prepared from the adults which were maintained by sibling mating for 12 generations. Illumina paired-end library with insert size of 300–500 bp was constructed and sequenced based on Illumina HiSeq X10 platform. Totally, 37 Gb Illumina HiSeq X10 reads were generated. The K-mer spectra, genome heterozygosity, and repeat content were generated using Jellyfish (version 1.1.10) with default parameters [[1](#_ENREF_1)].

**Nanopore library construction and sequencing**

Genome DNA was extracted from the adult insects using TIANamp Genomics DNA Kit (dp304, TIANGEN, Beijing, China). For each Nanopore library, ~8 ug gDNA was size-selected (10–50 kb) with Blue Pippin (Sage Science, Beverly, USA) and processed using the Ligation sequencing 1 d Kit (SQK-LSK108, Oxford Nanopore Technologies, Oxford, UK) according to the instructions. Totally 19 libraries were constructed and sequenced on 19 different R9.4 Flow Cells using Nanopore GridION X5 sequencer for 48 h each at the Genome Center of Nextomics (Wuhan, China). We use Oxford Nanopore Technologies Albacore software (version 0.8.4) (https://github.com/Albacore/albacore) to finish the base calling (Table S2).

**High-throughput chromosome conformation capture (Hi-C) library construction and sequencing**

We use Hi-C to assist chromosomal-scale genome assembly. The sample crosslinking was prepared as follows: ~1000 2–3 instar nymphs were cut with scissors, and then 1.25 ml 37% formaldehyde was added to obtain 2% final concentration and incubated for 10 min on plate at room temperature for further crosslink. After that, the crosslink was quenched by adding 2.5 ml 2.5 M glycine (incubated for 5 min at room temperature and then incubated on ice for 15 min). Finally, the samples were centrifuged at 4℃ with 2000 g for 10 min and the supernatant was removed. After the crosslinking, the samples were used for Hi-C library preparation and further sequenced using Illumina HiSeq X10 platform (Table S2).

**Hi-C scaffolding and chromosome assembly**

The Hi-C reads were uniquely mapped to the contig assembly and we retained the reads within 500 bp *HindIII* restriction regions for next analysis. We used 3D-DNA pipeline to correct the mis-joined contigs [[2](#_ENREF_2)]. ALLHiC pipeline was used to link the Hi-C corrected contigs into 10 pseudo-chromosomes [[3](#_ENREF_3)]. The accuracy of the Hi-C based assembly was evaluated by chromatin contact matrix (Figure S2).

**Mitochondrial genome assembly**

To assemble the mitochondrial sequences, we performed a reference-guided mitochondrion genome assembly strategy according to the recent published methods [[4](#_ENREF_4),[5](#_ENREF_5)]. We first selected the Nanopore reads with length ranging from 5 kb to 16 kb, and then aligned these reads to the national center for biotechnology information (NCBI) insect mitochondrial references using minimap2 [[6](#_ENREF_6)] and BLAST [[7](#_ENREF_7)]. The resulting MT reads were assembled using Canu v2.0 [[8](#_ENREF_8)], and contigs were aligned to the published reference (*Empoasca vitis* mitochondrion, NCBI accession number: NC_024838.1) [[9](#_ENREF_9)] by BLAST. MT contig was remain with highest identity and coverage. To polished this contig, we used corrected long reads by Racon and Medaka and short reads by freebayes [[10](#_ENREF_10)] combined with bcftools [[11](#_ENREF_11)]. The overlapping sequences at the both ends of contig were trimmed according self-alignment using nucmer [[12](#_ENREF_12)], and the remaining sequences is the finally circularized mitochondrial genome. Moreover, annotation was performed on the Goseq online platform using *E*. *vitis* mitochondrion as reference and with default parameters [[13](#_ENREF_13)].

**Repeat annotation**

A customized *de novo* repeat library was built using RepeatModeler (<http://www.repeatmasker.org/RepeatModeler/>), which recruits 2 repeat finding programs including RECON (version 1.08) and RepeatSout (version 1.0.5) [[14](#_ENREF_14)]. The generated transposable element (TE) consensus TE sequences were imported into the RepeatMasker (version 4.05) (http://www.repeatmasker.org) to further identify the repetitive elements. The unknown repeats were classified by TEclass (version 2.1.3) [[15](#_ENREF_15)]. Tandem Repeat Finder (TRF) (version 4.07) [[16](#_ENREF_16)] was used to identify the tandem repeats in the genome with the parameters: 1 1 2 80 5 200 2000 –d –h.

**Sample preparation for ribonucleic acid sequencing (RNA-Seq)**

We collected the samples of different developmental stages (eggs, nymphs, and adults). Besides, we also collected the insect samples from resistance and susceptible tea plants. Total RNA was extracted from all these samples using Trizol following the instructions and DNA contamination was removed using RNAase-Free DNase I (2270A, Takara, Beijing, China). These extracted RNA were sequenced on Illumina HiSeq X10 platform, yielding ~3 Gb data for each sample.

**Polymorphism (SNP) Calling**

A total of 57 **s**amples were collected from 4 tea regions (Jiangnan / South Central, Jiangbei / North Central, South China, and Southwest China) of China in this study. The 150-bp paired-end Illumina reads were yielded with the average coverage ranging from 20.1 × to 30.6 × (Table S12). Quality filtering was performed to remove adapters and low quality bases (Q < 30). Filtered reads were aligned against the reference genome using bwa with the default parameters. Variants were detected using the GATK (version: 3.5-0-g36282e4) [[17](#_ENREF_17)] following the best practices workflow for variant discovery. Then the IndelRealigner was used to locally realign the resulting BAM file to remove the mismatches located around small-scale deletions and insertions. Variants were called and merged using HaplotypeCaller and GenotypeGVCFs separately. This two-step approach includes quality recalibration and re-genotyping in the merged vcf file, which can ensure the variant accuracy. Meanwhile, samtools/bcftools were applied for SNPs calling using the same data with default parameters. SNPs were further filtered based on the workflow below: (1) SNPs were filtered if existed in only one of the two pipelines (GATK or samtools/bcftools); (2) SNPs located in repeat regions; (3) SNPs with high (> 1000) read depth or low reads depth (< 5); (4) SNPs with missing rate > 40%; (5) non-biallelic SNPs were removed; (6) SNPs with < 5bp distance with nearby variant sites.

**References**

[1] Marcais G, Kingsford C. A fast, lock-free approach for efficient parallel counting of occurrences of k-mers*.* Bioinformatics 2011;27:764–70.

[2] Dudchenko O, Batra SS, Omer AD, Nyquist SK, Hoeger M, Durand NC, et al. *De novo* assembly of the *Aedes aegypti* genome using Hi-C yields chromosome-length scaffolds*.* Science 2017;356:92–5.

[3] Zhang X, Zhang S, Zhao Q, Ming R, Tang H. Assembly of allele-aware, chromosomal-scale autopolyploid genomes based on Hi-C data*.* Nat Plants 2019;5:833–45.

[4] Rhie A, McCarthy SA, Fedrigo O, Damas J, Formenti G, Koren S et al. Towards complete and error-free genome assemblies of all vertebrate species*.* Nature 2021;592:737–46.

[5] Formenti G, Rhie A, Balacco J, Haase B, Mountcastle J, Fedrigo O, et al. Complete vertebrate mitogenomes reveal widespread repeats and gene duplications*.* Genome Biol 2021;22:120.

[6] Li H. Minimap2: pairwise alignment for nucleotide sequences*.* Bioinformatics 2018;34:3094–100.

[7] Altschul SF, Gish W, Miller W, Myers EW, Lipman DJ. Basic local alignment search tool*.* J Mol Biol 1990;215:403–10.

[8] Koren S, Walenz BP, Berlin K, Miller JR, Bergman NH, Phillippy AM. Canu: scalable and accurate long-read assembly via adaptive k-mer weighting and repeat separation*.* Genome Res 2017;27:722–36.

[9] Zhou N, Wang M, Cui L, Chen X, Han B. Complete mitochondrial genome of *Empoasca vitis* (Hemiptera: Cicadellidae)*.* Mitochondrial DNA A DNA Mapp Seq Anal 2016;27:1052–3.

[10] Garrison E, Marth G. Haplotype-based variant detection from short-read sequencing*.* arXiv 2012; https://doi.org/10.48550/arXiv.1207.3907.

[11] Danecek P, Auton A, Abecasis G, Albers CA, Banks E, DePristo MA, et al. The variant call format and VCFtools*.* Bioinformatics 2011;27:2156–8.

[12] Marcais G, Delcher AL, Phillippy AM, Coston R, Salzberg SL, Zimin A. MUMmer4: A fast and versatile genome alignment system*.* PLoS Comput Biol 2018;14:e1005944.

[13] Tillich M, Lehwark P, Pellizzer T, Ulbricht-Jones ES, Fischer A, Bock R, et al. GeSeq - versatile and accurate annotation of organelle genomes*.* Nucleic Acids Res 2017;45:W6–11.

[14] Price AL, Jones NC, Pevzner PA. *De novo* identification of repeat families in large genomes*.* Bioinformatics 2005;21:i351–8.

[15] Abrusan G, Grundmann N, DeMester L, Makalowski W. TEclass ― a tool for automated classification of unknown eukaryotic transposable elements*.* Bioinformatics 2009;25:1329–30.

[16] Benson G. Tandem repeats finder: a program to analyze DNA sequences*.* Nucleic Acids Res 1999;27:573–80.

[17] McKenna A, Hanna M, Banks E, Sivachenko A, Cibulskis K, Kernytsky A, et al. The Genome Analysis Toolkit: a MapReduce framework for analyzing next-generation DNA sequencing data*.* Genome Res 2010;20:1297–303.
