# Supplementary material for: Genomic Variations in the Tea Leafhopper Reveal the Basis of Its Adaptive Evolution
Source: Genomics Proteomics Bioinformatics. 2022 Aug 28;20(6):1092–105. doi: 10.1016/j.gpb.2022.05.011 (PMC10225489; doi:10.1016/j.gpb.2022.05.011)
Supplement: Supplementary Table S1 — Statistics of genomic sequencing data of E. onukii [file mmc2.docx]

**Table S1 Statistics of genomic sequencing data of *E*. *onukii***

| **Items** | **ONT** | **Illumina X10** |
| --- | --- | --- |
| Total number of reads (million) | 4 | 122 |
| Total number of sequenced bases (Gb) | 65 | 37 |
| Mean reads length (bp) | 15,737 | 150 |
| N50 (bp) | 24,965 | 150 |
| Coverage (×) | 109 | 61 |
