# Supplementary material for: Genomic Variations in the Tea Leafhopper Reveal the Basis of Its Adaptive Evolution
Source: Genomics Proteomics Bioinformatics. 2022 Aug 28;20(6):1092–105. doi: 10.1016/j.gpb.2022.05.011 (PMC10225489; doi:10.1016/j.gpb.2022.05.011)
Supplement: Supplementary Table S2 — Statistics of Hi-C mapping [file mmc3.docx]

**Table S2. Statistics of Hi-C mapping**

| **Statistics of mapping** | | |  |
| --- | --- | --- | --- |
| Clean Paired-end Reads | | 123,506,761 |  |
| Unmapped Paired-end Reads | | 52,279,737 |  |
| Unmapped Paired-end Reads Rate (%) | | 42.33 |  |
| Paired-end Reads with Singleton | | 53,084,389 |  |
| Paired-end Reads with Singleton Rate (%) | | 42.98 |  |
| Multi Mapped Paired-end Reads | | 4,576,854 |  |
| Multi Mapped Ratio (%) | | 3.71 |  |
| Unique Mapped Paired-end Reads | | 13,565,781 |  |
| Unique Mapped Ratio (%) | | 10.98 |  |
| **Statistics of valid reads** | | |  |
| Unique Mapped Paired-end Reads | 13,565,781 | |  |
| Dangling End Paired-end Reads | 6,409,964 | |  |
| Dangling End Rate (%) | 42.25 | |  |
| Self Circle Paired-end Reads | 95,862 | |  |
| Self Circle Rate (%) | 0.71 | |  |
| Dumped Paired-end Reads | 808,192 | |  |
| Dumped Rate (%) | 5.96 | |  |
| Interaction Paired-end Reads | 5,736,909 | |  |
| Interaction Rate (%) | 42.29 | |  |
| Lib Valid Paired-end Reads | 4,225,791 | |  |
| Lib Valid Rate (%) | 31.15 | |  |
| Lib Dup (%) | 26.34 | |  |
