# Supplementary material for: Genomic Variations in the Tea Leafhopper Reveal the Basis of Its Adaptive Evolution
Source: Genomics Proteomics Bioinformatics. 2022 Aug 28;20(6):1092–105. doi: 10.1016/j.gpb.2022.05.011 (PMC10225489; doi:10.1016/j.gpb.2022.05.011)
Supplement: Supplementary Table S3 — Statistics of contig level assembly of E. onukii [file mmc4.docx]

**Table S3 Statistics of contig level assembly of *E*. *onukii***

| Items | SMART-denovo | Wtdbg2 | Quickmerge |
| --- | --- | --- | --- |
| No. of contigs | 2568 | 2420 | 1800 |
| Max length (Mb) | 2.4 | 5.7 | 12,942,971 |
| Assembly size (Mb) | 637.8 | 571.8 | 599.1 |
| N90 (bp) | 99,924 | 162,530 | 388,364 |
| N50 (bp) | 410,283 | 1,134,910 | 2,172,144 |
| Average (bp) | 248,351 | 236,266 | 332,835 |
| Complete BUSCO ratio (%) | 95.3 | 91.5 | 92.7 |
| Duplicated BUSCO ratio (%) | 3.4 | 1.4 | 2.7 |
