# Supplementary material for: Genomic Variations in the Tea Leafhopper Reveal the Basis of Its Adaptive Evolution
Source: Genomics Proteomics Bioinformatics. 2022 Aug 28;20(6):1092–105. doi: 10.1016/j.gpb.2022.05.011 (PMC10225489; doi:10.1016/j.gpb.2022.05.011)
Supplement: Supplementary Table S4 — BUSCO analysis of genome assembly of E. onukii [file mmc5.docx]

**Table S4 BUSCO analysis of genome assembly of *E*. *onukii***

| **Description** | **Number** | **Percentage (%)** |
| --- | --- | --- |
| Complete BUSCOs | 1537 | 92.7 |
| Complete and single-copy BUSCOs (S) | 1493 | 90.0 |
| Complete and duplicated BUSCOs (D) | 44 | 2.7 |
| Fragmented BUSCOs (F) | 39 | 2.4 |
| Missing BUSCOs (M) | 82 | 4.9 |
| Total BUSCO groups searched | 1658 | 100 |
