# Supplementary material for: Genomic Variations in the Tea Leafhopper Reveal the Basis of Its Adaptive Evolution
Source: Genomics Proteomics Bioinformatics. 2022 Aug 28;20(6):1092–105. doi: 10.1016/j.gpb.2022.05.011 (PMC10225489; doi:10.1016/j.gpb.2022.05.011)
Supplement: Supplementary Table S5 — BUSCO analysis of annotation completeness [file mmc6.docx]

**Table S5 BUSCO analysis of annotation completeness**

| **Description** | **Number** | **Percentage (%)** |
| --- | --- | --- |
| Complete BUSCOs (C) | 1533 | 92.5 |
| Complete and single-copy BUSCOs (S) | 1462 | 88.2 |
| Complete and duplicated BUSCOs (D) | 71 | 4.3 |
| Fragmented BUSCOs (F) | 50 | 3.0 |
| Missing BUSCOs (M) | 75 | 4.5 |
| Total BUSCO groups searched | 1658 | 100 |
