# Supplementary material for: Genomic Variations in the Tea Leafhopper Reveal the Basis of Its Adaptive Evolution
Source: Genomics Proteomics Bioinformatics. 2022 Aug 28;20(6):1092–105. doi: 10.1016/j.gpb.2022.05.011 (PMC10225489; doi:10.1016/j.gpb.2022.05.011)
Supplement: Supplementary Table S6 — The statistics of different Hemiptera species assemblies [file mmc7.docx]

**Table S6 The statistics of different Hemiptera species assemblies**

| **Species** | **Chromosome level** | **Genome assembly size (Mb)** | **Estimated genome size (Mb)** | **Contig N50 (Kb)** | **Scaffold N50 (Kb)** |
| --- | --- | --- | --- | --- | --- |
| *A. pisum* | Yes | 464.3 | 512.2 | 10.8 | 22.8 |
| *A. glycines* | No | 302.9 | 317.2 | 15.8 | 174.5 |
| *D. noxia* | No | 393.0 | 417.2 | 12.6 | 397.8 |
| *M. persicae* | No | 347/355 | 409.3 | - | 435.8/164.5 |
| *N. lugens* | No | 1141 | 1220 | 24.2 | 356.6 |
| *O. fasciatus* | Yes | 1099 | 926 | 4.0 | 340.0 |
| *E. onukii* | Yes | 599.0 | 608.0 | 2200 | 67,980 |
