# Supplementary material for: Genomic Variations in the Tea Leafhopper Reveal the Basis of Its Adaptive Evolution
Source: Genomics Proteomics Bioinformatics. 2022 Aug 28;20(6):1092–105. doi: 10.1016/j.gpb.2022.05.011 (PMC10225489; doi:10.1016/j.gpb.2022.05.011)
Supplement: Supplementary Table S7 — Assessment of genome consistency based on NGS (Illumina) reads [file mmc8.docx]

**Table S7 Assessment of genome consistency based on NGS (Illumina) reads**

| **Items** | **Statistics** |
| --- | --- |
| Number of reads | 365,842,504 |
| Data size (Gb) | 54.88 |
| Mapped bases (Gb) | 50.64 |
| Mapping rate (%) | 92.27 |
| Genome length (Mb) | 599 |
| Mean depth | 78.65 |
| Coverage rate (%) | 93.80 |
