# Supplementary material for: Genomic Variations in the Tea Leafhopper Reveal the Basis of Its Adaptive Evolution
Source: Genomics Proteomics Bioinformatics. 2022 Aug 28;20(6):1092–105. doi: 10.1016/j.gpb.2022.05.011 (PMC10225489; doi:10.1016/j.gpb.2022.05.011)
Supplement: Supplementary Table S8 — Statistics of TEs in E. onukii genome [file mmc9.docx]

**Table S8 Statistics of TEs in *E*. *onukii* genome**

|  | **Number** | **Length (bp)** | **% of repeats** | **% of genome** |
| --- | --- | --- | --- | --- |
| **Total repeat fraction** | 1,153,567 | 245,482,504 | 100 | 38.44 |
| **Class I: retroelement** | | | | |
| **LTR retrotransposon** | 143,294 | 25,581,399 | 10.62 | 4.00 |
| Ty1/Copia | 116 | 90,347 | 0.04 | 0.01 |
| Ty3/Gypsy | 2801 | 1,542,498 | 0.64 | 0.24 |
| Other | 140,377 | 23,948,554 | 9.94 | 3.75 |
| **Non-LTR retrotransposon** | 337,969 | 85,834,974 | 35.63 | 13.44 |
| LINE | 242,812 | 71,768,330 | 29.79 | 11.24 |
| SINE | 95,157 | 14,066,644 | 5.84 | 2.20 |
| **Unclassified retroelement** | 154,284 | 26,124,809 | 10.84 | 4.09 |
| **Class II: DNA transposon** | | | | |
| **TIR** | 353,180 | 72,713,311 | 30.18 | 11.38 |
| hAT | 8857 | 1,563,092 | 0.65 | 0.24 |
| Mutator | 353 | 80,791 | 0.03 | 0.01 |
| Tc1/Mariner | 30,632 | 9,167,282 | 3.81 | 1.44 |
| PIF/Harbinger | 5459 | 756,136 | 0.31 | 0.12 |
| Other | 307,879 | 61,146,010 | 25.38 | 9.57 |
| **Helitron** | 15,028 | 3,675,120 | 1.53 | 0.58 |
| **Tandem repeats** | 100,545 | 20,953,073 | 8.70 | 3.28 |
| **Unknown** | 49,267 | 10,599,818 | 4.40 | 1.66 |

*Note*: TEs, Transposable elements; LTR, long terminal repeat; LINE, long interspersed nuclear elements; SINE, short interspersed nuclear elements; TIR, terminal inverted repeat; PIF, P Instability Factor.
