# Supplementary material for: Genomic Variations in the Tea Leafhopper Reveal the Basis of Its Adaptive Evolution
Source: Genomics Proteomics Bioinformatics. 2022 Aug 28;20(6):1092–105. doi: 10.1016/j.gpb.2022.05.011 (PMC10225489; doi:10.1016/j.gpb.2022.05.011)
Supplement: Supplementary Table S10 — Gene family contraction analysis on E. onukii branch [file mmc11.docx]

**Table S10 Gene family contraction analysis on *E*. *onukii* branch**

| **Pfam domain** | **Number of genes in each species of hemipteran** | | | | |
| --- | --- | --- | --- | --- | --- |
|  | ***N*. *lugens*** | ***A*. *pisum*** | ***B*. *t* *abaci*** | ***E*. *onukii*** | ***M*. *persicae*** |
| Immunoglobulin | 250 | 365 | 295 | 205 | 335 |
| Myosin | 78 | 92 | 84 | 22 | 93 |
| Tropomyosin | 22 | 31 | 50 | 10 | 30 |
