# Supplementary material for: Genomic Variations in the Tea Leafhopper Reveal the Basis of Its Adaptive Evolution
Source: Genomics Proteomics Bioinformatics. 2022 Aug 28;20(6):1092–105. doi: 10.1016/j.gpb.2022.05.011 (PMC10225489; doi:10.1016/j.gpb.2022.05.011)
Supplement: Supplementary Table S13 — Geographic distributions and population structure of the collected samples around China [file mmc14.docx]

**Table S13 Geographic distributions of the collected samples around China**

| **IDs** | **Location** | **Province** | **Longitude** | **Latitude** | **Tea regions** | **Group** | **Clean data** | **Coverage (×)** | **Mapping rate** | **SNPs** | **Indels** |
| --- | --- | --- | --- | --- | --- | --- | --- | --- | --- | --- | --- |
| 1 | ShaoYang | HuNan | 110.4883333 | 27.05944444 | SYR | Group III | 13.0G | 21.8 | 98.21% | 15,154,757 | 4,280,186 |
| 2 | Wuyishan | FuJian | 118.0017889 | 27.71386389 | SER | Group III | 14.1G | 23.7 | 98.03% | 16,212,801 | 4,648,417 |
| 3 | SuiChuan | JiangXi | 114.2602583 | 26.09143611 | SYR | Group III | 13.9G | 23.3 | 98.14% | 16,130,403 | 4,622,915 |
| 4 | Sheshan | ShangHai | 121.1866306 | 31.09467222 | SYR | Group II | 14.4G | 24.2 | 98.25% | 15,858,581 | 4,530,022 |
| 5 | WuYi | ZheJiang | 119.7913472 | 28.89059444 | SYR | Group II | 16.0G | 26.9 | 98.17% | 15,891,379 | 4,589,949 |
| 6 | AnXi | FuJian | 117.8739722 | 25.00241667 | SER | Group III | 15.2G | 25.5 | 98.22% | 16,809,151 | 4,845,517 |
| 7 | DouJun | GuiZhou | 107.4756139 | 26.35382222 | SWR | Group III | 16.0G | 26.9 | 98.21% | 16,511,092 | 4,760,991 |
| 8 | FengGang | GuiZhou | 107.6997417 | 28.02914444 | SWR | Group III | 15.2G | 25.5 | 98.20% | 16,802,849 | 4,831,110 |
| 9 | LinHai | ZheJiang | 121.141831 | 28.977566 | SYR | Group II | 15.7G | 26.4 | 98.26% | 16,669,852 | 4,767,208 |
| 10 | XiHu | ZheJiang | 120.0911333 | 30.18467222 | SYR | Group II | 13.7G | 23.0 | 94.71% | 15,232,069 | 4,291,995 |
| 11 | NanNing | GuangXi | 109.1472528 | 22.75567778 | SER | Group III | 16.2G | 27.2 | 98.65% | 16,029,598 | 4,601,254 |
| 12 | PuJiang | SiChuan | 103.3807472 | 30.16399444 | SWR | Group III | 15.7G | 26.4 | 97.86% | 15,893,642 | 4,553,711 |
| 13 | TengChong | YunNan | 98.67929167 | 24.92432222 | SWR | Group I | 15.7G | 26.4 | 98.21% | 12,215,935 | 3,516,260 |
| 14 | WuZhiShan | HeNan | 109.5899972 | 18.68604167 | SER | Group III | 16.8G | 28.2 | 98.67% | 16,016,362 | 4,610,438 |
| 15 | GanZhou | GuangDong | 116.7000694 | 23.80833611 | SER | Group II | 15.8G | 26.5 | 98.32% | 14,877,178 | 4,294,313 |
| 16 | PuEr | YunNan | 100.9223944 | 22.76429167 | SWR | Group I | 18.2G | 30.6 | 90.75% | 14,011,492 | 4,010,930 |
| 17 | ShiYan | HuNan | 110.4883333 | 27.05944444 | SYR | Group III | 16.7G | 28.0 | 98.31% | 16,623,326 | 4,772,514 |
| 18 | YuXi | YunNan | 102.2699194 | 24.146275 | SWR | Group I | 15.2G | 25.5 | 98.04% | 14,805,955 | 4,235,716 |
| 19 | MoTuo | XiZang | 95.35646667 | 29.192725 | SWR | Group III | 14.7G | 24.7 | 98.38% | 15,182,502 | 4,340,031 |
| 20 | MianYang | SiChuan | 104.4594361 | 31.81407222 | SWR | Group III | 14.7G | 24.7 | 98.26% | 15,233,861 | 4,346,004 |
| 21 | ChangSha | HuNan | 113.2686111 | 28.30611111 | SYR | Group III | 13.7G | 23.0 | 97.80% | 15,334,791 | 4,365,402 |
| 22 | GuZhang | HuNan | 109.8780556 | 28.62 | SYR | Group III | 14.5G | 24.3 | 98.33% | 15,902,090 | 4,546,546 |
| 23 | EnShi | HuBei | 109.5027778 | 30.06444444 | SYR | Group III | 15.8G | 26.5 | 98.04% | 16,180,528 | 4,626,792 |
| 24 | WuHan | HuBei | 114.5027778 | 30.99611111 | SYR | Group III | 16.5G | 27.7 | 98.25% | 16,269,457 | 4,657,863 |
| 25 | LuShan | JiangXi | 115.875 | 29.50486389 | SYR | Group III | 15.7G | 26.4 | 98.37% | 16,554,166 | 4,754,175 |
| 26 | BeiFeng | FuJian | 119.3872528 | 26.153125 | SER | Group III | 16.7G | 28.0 | 98.31% | 17,021,243 | 4,898,709 |
| 27 | QingYuan | GuangDong | 113.2948111 | 24.33394722 | SER | Group III | 15.3G | 25.7 | 97.48% | 16,020,286 | 4,592,591 |
| 28 | GuiLin | GuangXi | 110.3541167 | 25.29503611 | SER | Group III | 15.7G | 26.4 | 98.28% | 15,905,714 | 4,557,483 |
| 29 | ZhanJiang | GuangDong | 110.2488778 | 20.51934444 | SER | Group III | 15.5G | 26.0 | 98.29% | 15,711,797 | 4,485,127 |
| 30 | BaiSe | GuangXi | 106.580225 | 24.46293889 | SER | Group III | 15.9G | 26.7 | 97.49% | 13,320,883 | 3,707,926 |
| 31 | FuDing | FuJian | 120.1327528 | 27.22771389 | SER | Group II | 12.2G | 20.5 | 98.43% | 15,015,367 | 4,221,552 |
| 32 | XiXiang | Shaanxi | 107.6333667 | 32.89587222 | NYR | Group III | 14.1G | 23.7 | 96.93% | 15,117,055 | 4,304,592 |
| 33 | XinZhu | TaiWan | 121.0275 | 25.19277778 | SER | Group II | 16.2G | 27.2 | 98.17% | 15,739,439 | 4,524,321 |
| 34 | NanTou | TaiWan | 121.0502778 | 24.05416667 | SER | Group II | 15.0G | 25.2 | 98.34% | 14,478,659 | 4,165,842 |
| 35 | XiShan | NeNan | 111.0791361 | 33.47525278 | NYR | Group III | 13.5G | 22.7 | 97.63% | 15,381,268 | 4,364,716 |
| 36 | NanChang | JiangXi | 116.0043611 | 28.37004722 | SYR | Group II | 15.4G | 25.9 | 98.56% | 16,502,613 | 4,703,669 |
| 37 | ZhouShan | ZheJiang | 122.0748472 | 30.08829167 | SYR | Group III | 13.3G | 22.3 | 98.35% | 15,396,559 | 4,367,697 |
| 38 | HaiKou | HaiNan | 110.4597611 | 19.46704444 | SER | Group III | 16.0G | 26.9 | 98.26% | 15,219,466 | 4,369,003 |
| 39 | LianYunGang | JiangSu | 119.3177167 | 34.66125 | SYR | Group II | 15.1G | 25.3 | 98.39% | 15,988,727 | 4,558,834 |
| 40 | QiMen | AnHui | 117.5133139 | 29.84614167 | SYR | Group II | 13.3G | 22.3 | 97.36% | 15,055,123 | 4,276,018 |
| 41 | FengYang | AnHui | 117.8724722 | 32.65033611 | SYR | Group III | 17.3G | 29.0 | 97.62% | 16,678,678 | 4,776,883 |
| 42 | LiuAn | AnHui | 116.2199889 | 31.39157778 | SYR | Group III | 14.3G | 24.0 | 98.57% | 15,956,534 | 4,535,057 |
| 43 | KaiHua | ZheJiang | 118.2636028 | 29.11968333 | SYR | Group II | 16.9G | 28.4 | 98.66% | 16,037,216 | 4,599,723 |
| 44 | Nairobi | Africa | 36.49 | 1.17 | Africa | outgroup | 13.1G | 22.0 | 33.89% | 29,019 | 4749 |
| 45 | JiYuan | HeNan | 112.4295694 | 35.191525 | NYR | Group II | 13.8G | 23.2 | 98.57% | 14,738,189 | 4,185,898 |
| 46 | XinYang | HeNan | 113.7857583 | 32.19541389 | NYR | Group III | 12.4G | 20.8 | 98.56% | 15,018,422 | 4,238,150 |
| 47 | LongNan | GanSu | 105.2887 | 32.73546944 | NYR | Group III | 16.1G | 27.0 | 96.25% | 15,877,287 | 4,544,245 |
| 48 | MengHai | YunNan | 100.4997083 | 21.79693889 | SWR | Group I | 13.5G | 22.7 | 98.27% | 11,055,619 | 3,150,374 |
| 49 | ZiYang | ShanXi | 108.5241639 | 32.52690833 | NYR | Group III | 15.0G | 25.2 | 92.10% | 13,869,412 | 3,950,991 |
| 50 | TaiShan | ShanDong | 117.1619167 | 36.27371667 | NYR | Group II | 18.3G | 30.7 | 80.30% | 13,580,586 | 3,815,772 |
| 51 | SuZhou | JiangSu | 120.3835833 | 31.09733333 | SYR | Group II | 20.7G | 34.7 | 91.89% | 16,483,588 | 4,744,757 |
| 52 | RiZhao | ShanDong | 119.2616833 | 35.31455 | NYR | Group II | 15.7G | 26.4 | 92.71% | 14,785,789 | 4,197,553 |
| 53 | LaoShan | ShanDong | 120.6853333 | 36.15211667 | NYR | Group II | 14.5G | 24.3 | 98.60% | 15,455,293 | 4,399,335 |
| 54 | TaiZHou | ZheJiang | 121.141831 | 28.977566 | SYR | Group II | 16.9G | 28.4 | 98.43% | 16,836,234 | 4,835,103 |
| 55 | Toronto | Canada | 79.25 | 43.4 | Canada | outgroup | 14.6G | 24.5 | 39.17% | 375,579 | 63,563 |
| 56 | LiShui | ZheJiang | 119.3290116 | 28.035737 | SYR | Group II | 15.0G | 25.2 | 98.33% | 16,439,489 | 4,685,444 |
| Total Number of high-quality SNPs | | | | | | | | | | 12,271,501 | |

*Note*: SWR, southwest region; SYR, south of the Yangtze River region; NYR, north of the Yangtze River region; SER, south China region.
