# Supplementary material for: Genomic Variations in the Tea Leafhopper Reveal the Basis of Its Adaptive Evolution
Source: Genomics Proteomics Bioinformatics. 2022 Aug 28;20(6):1092–105. doi: 10.1016/j.gpb.2022.05.011 (PMC10225489; doi:10.1016/j.gpb.2022.05.011)
Supplement: Supplementary Table S16 — Functional analysis of genes under purifying selection [file mmc17.docx]

**Table S16 Functional analysis of genes under purifying selection**

| **Gene ID** | **Functional annotation** | **E-value** |
| --- | --- | --- |
| gene_10670-MYZPE13164_G006_V1.0_000135270.2_R1 | Myelin regulatory factor | 0 |
| gene_11818-RNA12909_R0 | Forkhead box protein P1 | 9E-151 |
| gene_15492-RNA10461_R0 | Coronin-6 | 0 |
| gene_15488-RNA19235_R0 | Inositol hexakisphosphate and Diphosphoinositol-pentakisphosphate kinase 2 | 0 |
| gene_15510-RNA2388_R0 | KICSTOR complex protein SZT2-like | 0 |
| gene_15507-RNA5810_R0 | Protein tyrosine phosphatase | 0 |
| gene_15477-GB41265-RA_R0 | Nucleoredoxin-like isoform | 2E-75 |
